# Intronic *FGF14* GAA repeat expansions impact progression and survival in multiple system atrophy

**DOI:** 10.1093/brain/awaf134

**Published:** 2025-04-16

**Authors:** Viorica Chelban, David Pellerin, Nirosen Vijiaratnam, Hamin Lee, Yen Yee Goh, Lauren Brown, Sara Sambin, Danielle Seilhean, Stephane Lehericy, Pablo Iruzubieta, Rahema Mohammad, Eleanor Self, Annarita Scardamaglia, Cameron Lee, Miriama Ostrozovicova, Marie-Josée Dicaire, Christine Girges, Emil K Gustavsson, David Murphy, Toby Curless, Joshua Laß, Joanne Trinh, Timothy Rittman, James B Rowe, Marios Hadjivassiliou, Neil Archibald, Matt C Danzi, Catherine Ashton, Virginie Roth, Marion Wandzel, Warren A Cheung, Djordje O Gveric, Bart De Vil, Jordan Follett, P Nigel Leigh, Lukas Beichert, Tomi Pastinen, Céline Bonnet, Mathilde Renaud, Wassilios G Meissner, Anne Sieben, David Crosiers, Patrick Cras, Stephan Zuchner, Jean-Christophe Corvol, Matthew J Farrer, Matthis Synofzik, Bernard Brais, Tom Warner, Huw R Morris, Zane Jaunmuktane, Tom Foltynie, Henry Houlden

**Affiliations:** Neuromuscular Disease Department, UCL Queen Square Institute of Neurology, University College London, London WC1N 3BG, UK; Neurobiology and Medical Genetics Laboratory, ‘Nicolae Testemitanu’ State University of Medicine and Pharmacy, Chisinau MD-2004, Republic of Moldova; Neuromuscular Disease Department, UCL Queen Square Institute of Neurology, University College London, London WC1N 3BG, UK; Dr. John T. Macdonald Foundation Department of Human Genetics and John P. Hussman Institute for Human Genomics, University of Miami Miller School of Medicine, Miami, FL 33136, USA; Department of Neurology and Neurosurgery, Montreal Neurological Hospital and Institute, McGill University, Montreal, QC H3A 2B4, Canada; Clinical Research Centre for Movement Disorders and Gait, Kingston Centre, Parkinson’s Foundation Centre of Excellence, Monash Health, Cheltenham, VIC 3192, Australia; Department of Medicine, School of Clinical Sciences, Monash University, Clayton, VIC 3168, Australia; Neuromuscular Disease Department, UCL Queen Square Institute of Neurology, University College London, London WC1N 3BG, UK; Neuromuscular Disease Department, UCL Queen Square Institute of Neurology, University College London, London WC1N 3BG, UK; Neuromuscular Disease Department, UCL Queen Square Institute of Neurology, University College London, London WC1N 3BG, UK; Institut du Cerveau–Paris BrainInstitute–ICM, Inserm, CNRS, Sorbonne Université, Paris 75651, France; Department of Neurology, Assistance Publique Hôpitaux de Paris, CIC Neurosciences, Hôpital Pitié-Salpêtrière, Paris 75651, France; Département de Neuropathologie, Institut de Neurologie, DMU Neurosciences, Groupe Hospitalier Pitié-Salpêtrière, 47–83 boulevard de l’Hôpital, Paris 75651, France; Institut du Cerveau–Paris BrainInstitute–ICM, Inserm, CNRS, Sorbonne Université, Paris 75651, France; Department of Neuroradiology, Assistance Publique Hôpitaux de Paris, Hôpital Pitié-Salpêtrière, Paris 75013, France; Neuromuscular Disease Department, UCL Queen Square Institute of Neurology, University College London, London WC1N 3BG, UK; Department of Neurology and Neurosurgery, Montreal Neurological Hospital and Institute, McGill University, Montreal, QC H3A 2B4, Canada; Department of Neurology, Donostia University Hospital, Biogipuzkoa Health Research Institute, Donostia-San Sebastián 20014, Spain; CIBERNED Centro de Investigación Biomédica en Red en Enfermedades Neurodegenerativas, Instituto de Salud Carlos III (CIBER-CIBERNED-ISCIII), Madrid 28029, Spain; Neuromuscular Disease Department, UCL Queen Square Institute of Neurology, University College London, London WC1N 3BG, UK; Neuromuscular Disease Department, UCL Queen Square Institute of Neurology, University College London, London WC1N 3BG, UK; Neuromuscular Disease Department, UCL Queen Square Institute of Neurology, University College London, London WC1N 3BG, UK; Neuromuscular Disease Department, UCL Queen Square Institute of Neurology, University College London, London WC1N 3BG, UK; Neuromuscular Disease Department, UCL Queen Square Institute of Neurology, University College London, London WC1N 3BG, UK; Department of Neurology and Neurosurgery, Montreal Neurological Hospital and Institute, McGill University, Montreal, QC H3A 2B4, Canada; Department Clinical and Movement Neuroscience, UCL Queen Square Institute of Neurology, University College London, London WC1N 3BG, UK; Genetics and Genomic Medicine, Great Ormond Street Institute of Child Health, University College London, London WC1N 3BG, UK; UK Dementia Research Institute, The University of Cambridge, Cambridge CB2 0AH, UK; Department Clinical and Movement Neuroscience, UCL Queen Square Institute of Neurology, University College London, London WC1N 3BG, UK; Department Clinical and Movement Neuroscience, UCL Queen Square Institute of Neurology, University College London, London WC1N 3BG, UK; Queen Square Brain Bank for Neurological Disorders, UCL Queen Square Institute of Neurology, London WC1N 1PJ, UK; Institute of Neurogenetics, University of Lübeck, Lübeck 23538, Germany; Institute of Neurogenetics, University of Lübeck, Lübeck 23538, Germany; Department of Clinical Neurosciences, Cambridge University, Cambridge CB2 0SZ, UK; Department of Neurology, Cambridge University Hospitals NHS Trust, Cambridge CB2 0QQ, UK; Department of Clinical Neurosciences, Cambridge University, Cambridge CB2 0SZ, UK; Department of Neurology, Cambridge University Hospitals NHS Trust, Cambridge CB2 0QQ, UK; MRC Cognition and Brain Sciences Unit, Cambridge University, Cambridge CB2 7EF, UK; Academic Department of Neurosciences, Sheffield Teaching Hospitals NHS Trust, Royal Hallamshire Hospital, Sheffield S10 2JF, UK; Department of Neurology, South Tees NHS Foundation Trust, Middlesbrough TS4 3BW, UK; Dr. John T. Macdonald Foundation Department of Human Genetics and John P. Hussman Institute for Human Genomics, University of Miami Miller School of Medicine, Miami, FL 33136, USA; Department of Neurology and Neurosurgery, Montreal Neurological Hospital and Institute, McGill University, Montreal, QC H3A 2B4, Canada; Department of Neurology, Royal Perth Hospital, Perth, WA 6000, Australia; Laboratoire de Génétique, CHRU de Nancy, Nancy 54511, France; Laboratoire de Génétique, CHRU de Nancy, Nancy 54511, France; Genomic Medicine Center, Children’s Mercy Kansas City, Kansas City, MO 64108, USA; Department of Brain Sciences, Imperial College London, London W12 0NN, UK; Neuropathology Lab, IBB-NeuroBiobank BB190113, Born Bunge Institute, Antwerp 2610, Belgium; Faculty of Medicine and Health Sciences, Translational Neurosciences, Born-Bunge Institute, University of Antwerp, Antwerp 2610, Belgium; Department of Neurology, Antwerp University Hospital, Edegem 2650, Belgium; Department of Neurology, McKnight Brain Institute, University of Florida, Florida, FL 32610, USA; Department of Neuroscience, Brighton and Sussex Medical School, Brighton BN1 9PX, UK; German Center for Neurodegenerative Diseases (DZNE), Tübingen 72076, Germany; Division of Translational Genomics of Neurodegenerative Diseases, Hertie-Institute for Clinical Brain Research and Center of Neurology, University of Tübingen, Tübingen 72076, Germany; Service de Génétique Clinique, CHRU de Nancy, Nancy 54511, France; Laboratoire de Génétique, CHRU de Nancy, Nancy 54511, France; INSERM-U1256 NGERE, Université de Lorraine, Nancy 54500, France; Service de Génétique Clinique, CHRU de Nancy, Nancy 54511, France; INSERM-U1256 NGERE, Université de Lorraine, Nancy 54500, France; Service de Neurologie, CHRU de Nancy, Nancy 54511, France; Service de Neurologie-Maladies Neurodégénératives, IMNc, CRMR AMS, CHU de Bordeaux, Bordeaux 33076, France; CNRS, IMN, UMR 5293, University of Bordeaux, Bordeaux F-33000, France; Department of Medicine, University of Otago, Christchurch 9016, New Zealand; New Zealand Brain Research Institute, Christchurch 8011, New Zealand; Neuropathology Lab, IBB-NeuroBiobank BB190113, Born Bunge Institute, Antwerp 2610, Belgium; Faculty of Medicine and Health Sciences, Translational Neurosciences, Born-Bunge Institute, University of Antwerp, Antwerp 2610, Belgium; Department of Pathology, Antwerp University Hospital-UZA, Antwerp 2650, Belgium; Faculty of Medicine and Health Sciences, Translational Neurosciences, Born-Bunge Institute, University of Antwerp, Antwerp 2610, Belgium; Department of Neurology, Antwerp University Hospital, Edegem 2650, Belgium; Faculty of Medicine and Health Sciences, Translational Neurosciences, Born-Bunge Institute, University of Antwerp, Antwerp 2610, Belgium; Department of Neurology, Antwerp University Hospital, Edegem 2650, Belgium; Dr. John T. Macdonald Foundation Department of Human Genetics and John P. Hussman Institute for Human Genomics, University of Miami Miller School of Medicine, Miami, FL 33136, USA; Institut du Cerveau–Paris BrainInstitute–ICM, Inserm, CNRS, Sorbonne Université, Paris 75651, France; Department of Neurology, Assistance Publique Hôpitaux de Paris, CIC Neurosciences, Hôpital Pitié-Salpêtrière, Paris 75651, France; Department of Neurology, McKnight Brain Institute, University of Florida, Florida, FL 32610, USA; German Center for Neurodegenerative Diseases (DZNE), Tübingen 72076, Germany; Division of Translational Genomics of Neurodegenerative Diseases, Hertie-Institute for Clinical Brain Research and Center of Neurology, University of Tübingen, Tübingen 72076, Germany; Department of Neurology and Neurosurgery, Montreal Neurological Hospital and Institute, McGill University, Montreal, QC H3A 2B4, Canada; Department of Human Genetics, McGill University, Montreal, QC H3A 0G4, Canada; Department Clinical and Movement Neuroscience, UCL Queen Square Institute of Neurology, University College London, London WC1N 3BG, UK; Queen Square Brain Bank for Neurological Disorders, UCL Queen Square Institute of Neurology, London WC1N 1PJ, UK; Department Clinical and Movement Neuroscience, UCL Queen Square Institute of Neurology, University College London, London WC1N 3BG, UK; Department Clinical and Movement Neuroscience, UCL Queen Square Institute of Neurology, University College London, London WC1N 3BG, UK; Queen Square Brain Bank for Neurological Disorders, UCL Queen Square Institute of Neurology, London WC1N 1PJ, UK; Division of Neuropathology, National Hospital for Neurology and Neurosurgery, University College London NHS Foundation Trust, London WC1N 3BG, UK; Department Clinical and Movement Neuroscience, UCL Queen Square Institute of Neurology, University College London, London WC1N 3BG, UK; Neuromuscular Disease Department, UCL Queen Square Institute of Neurology, University College London, London WC1N 3BG, UK

**Keywords:** multiple system atrophy, spinocerebellar ataxia 27B, *FGF14* GAA ataxia

## Abstract

Partial phenotypic overlap has been suggested between multiple system atrophy and spinocerebellar ataxia 27B, the autosomal dominant ataxia caused by an intronic GAA•TTC repeat expansion in *FGF14*. In this study, we investigated the frequency of *FGF14* GAA•TTC repeat expansion in clinically diagnosed and pathologically confirmed multiple system atrophy cases.

We screened 657 multiple system atrophy cases (193 clinically diagnosed and 464 pathologically confirmed) and 1003 controls. The *FGF14* repeat locus was genotyped using long-range PCR and bidirectional repeat-primed PCRs, and expansions were confirmed with targeted long-read Oxford Nanopore Technologies sequencing.

We identified 19 multiple system atrophy cases carrying an *FGF14* GAA_≥250_ expansion (2.89%, *n* = 19/657), a significantly higher frequency than in controls (1.40%, *n* = 12/1003) (*P* = 0.04). Long-read Oxford Nanopore Technologies sequencing confirmed repeat sizes and polymorphisms detected by PCR, with high concordance (Pearson's *r* = 0.99, *P* < 0.0001). Seven multiple system atrophy patients had a pathogenic *FGF14* GAA_≥300_ expansion (five pathologically confirmed and two clinically diagnosed), and 12 had intermediate GAA_250–299_ expansion (six pathologically confirmed and six clinically diagnosed). A similar proportion of cerebellar-predominant and parkinsonism-predominant multiple system atrophy cases had *FGF14* expansions. Multiple system atrophy patients carrying an *FGF14* GAA_≥250_ expansion exhibited severe gait ataxia, autonomic dysfunction and parkinsonism, in keeping with a multiple system atrophy phenotype, with a faster progression to falls (*P* = 0.03) and regular wheelchair use (*P* = 0.02) in comparison to the multiple system atrophy cases without *FGF14* GAA expansion. The length of the GAA•TTC repeat expansion lengths was inversely correlated with survival in multiple system atrophy patients (*r* = −0.67; *P* = 0.02) but not with age of onset.

Therefore, screening for *FGF14* GAA•TTC repeat expansion should be considered for multiple system atrophy patients with rapid loss of mobility and for complete diagnostic accuracy at inclusion in disease-modifying multiple system atrophy drug trials.

## Teaser

Chelban *et al.* screened more than 650 individuals with multiple system atrophy and found *FGF14* GAA•TTC repeat expansions in about 3% of cases. These expansions were associated with more rapid mobility loss and shorter survival. *FGF14* testing should be considered for complete diagnostic accuracy in trials of disease-modifying drugs.

## Introduction

Multiple system atrophy (MSA) is a rare adult-onset neurodegenerative disorder whose aetiology remains unknown. It is characterized by a variable combination of progressive parkinsonism, cerebellar ataxia and dysautonomia, although a predominance of either parkinsonism (MSA-P) or cerebellar impairment (MSA-C) usually occurs.^1^ The pathological hallmark of MSA is the accumulation of α-synuclein, which aggregates in mature oligodendrocytes to form glial cytoplasmic inclusions, defining it as a synucleinopathy alongside Parkinson's disease and dementia with Lewy bodies.^2,3^ Diagnosis of MSA can be challenging, with ∼80% of clinically diagnosed cases meeting pathological criteria at autopsy.^4-6^ Clinical heterogeneity at presentation and during progression partly explain the diagnostic challenges, although contributing factors, including genetic causes and modifiers, remain poorly understood. Although family history was an exclusion criterion in previous MSA diagnostic criteria,^4^ they have been revised to be inclusive.^7^ Multiplex families have been described with mixed MSA and Lewy body disease presentations linked to rare *COQ2* variants in Japanese families, although data supporting increased risk for MSA with common *COQ2* variants remains equivocal.^8,9^ Recently, more risk loci in MSA were identified implicating *GAB1*, *lnc-LRRC49-3*, *TENM2* and *RABGEF1* in Europeans^10^ and *PLA2G4C* in meta-analysis of Asian and Caucasian MSA patients.^11^

Spinocerebellar ataxia 27B (SCA27B), caused by an intronic GAA•TTC repeat expansion in the fibroblast growth factor 14 gene (*FGF14*),^12,13^ has emerged as a significant contributor to previously undiagnosed cases of late-onset cerebellar ataxia.^12,14,15^ Repeat expansions of ≥250 GAA•TTC repeats (GAA_≥250_) are considered pathogenic, albeit with incomplete penetrance for expansions of 250–299 repeat units.^12,13,15^ Symptom onset typically occurs in the fifth to seventh decade.^16^ Episodic ataxia and cerebellar ocular motor disturbances, such as downbeat nystagmus, are hallmark features of SCA27B, whereas non-cerebellar manifestations, including parkinsonism and dysautonomia, have been reported with variable frequency across different cohorts.^12,14,17,18^ The observation of features of neurogenic bladder and pyramidal signs in some patients, in addition to late-onset ataxia, also suggests partial phenotypic overlap between SCA27B and MSA.^18^ Thus we investigated the frequency of *FGF14* GAA•TTC repeat expansion in clinically diagnosed MSA patients and pathologically confirmed MSA cases and explored the diagnostic and prognostic implications.

## Materials and methods

Patients with MSA included in this study were recruited with informed consent under ethics-approved research protocols. The study was approved by institutional review boards of the University College London, London (UCLH: 04/N034), Montreal Neurological Hospital, Montreal (MPE-CUSM-15-915), the Center for Neurology, Tübingen (598/2011BO1) and the Children's Mercy Kansas City (Study #11120514).

### Subjects

**Figure 1:**
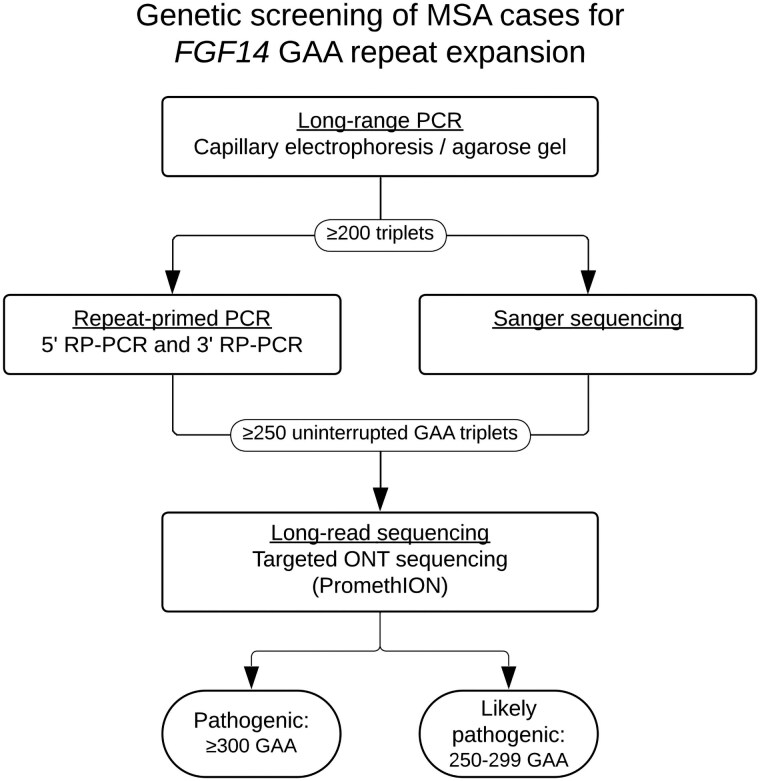
**Study flowchart diagram.** MSA = multiple system atrophy; ONT = Oxford Nanopore Technologies; RP-PCR = repeat-primed PCR.

The screening flowchart is illustrated in Fig. 1. Pathologically confirmed MSA patients were recruited as part of an international collaboration of movement disorders centres, brain banks and the cohort of patients from the PROSPECT-M UK study, the Neuroprotection and Natural History in Parkinson Plus Syndromes (NNIPPS) study^19^ (Supplementary material, ‘Data’ sections) and GIE-Neuro CEB (BB-0033-00011).

The definite neuropathological diagnosis of MSA was established based on MSA working group criteria.^20^ Definite MSA patients were characterized further as striatonigral degeneration (SND) or olivopontocerebellar atrophy (OPCA) predominant, based on autopsy findings.

Clinically diagnosed MSA patients were recruited from the MSA specialist clinic at UCL between 2012 and 2021 and fulfilled the diagnosis of probable MSA according to the 2008 MSA diagnostic criteria.^4^ The 2022 MSA diagnostic criteria^7^ were not applied, because patients were diagnosed prior to the publication. The 2022 MSA diagnostic criteria were applied in retrospect to all GAA_≥300_-*FGF14*-positive MSA cases, with all of them meeting the most updated criteria. In addition, lack of family history of cerebellar ataxia, disease onset after 30 years of age and negative genetic testing for common repeat expansions causing ataxia (expansions in *ATXN1*, *ATXN2*, *ATXN3*, *ATXN7*, *CACNA1A*, *TBP*, *ATN1*, *FXN*, *RFC1* and *FMR1*) was confirmed in all clinically diagnosed cases. Clinically diagnosed MSA patients were assigned ‘probable’ or ‘possible’ cerebellar (MSA-C), parkinsonian (MSA-P) or mixed (MSA-mixed) subtypes. There was no bias selection for any MSA clinical subtype at inclusion in this analysis. Participants reported their own sex, race and ethnic group.

Phenotyping was performed through a review of medical records and neuroimaging performed as part of standard clinical care (with variable acquisition protocol) and, when possible, patient re-evaluation using a standardized data sheet for all MSA with an *FGF14* expansion of ≥250 repeats.

We analysed disease milestones from a subset of patients who took part in a longitudinal MSA study as part of the Progressive Supranuclear Palsy–Corticobasal Syndrome–Multiple System Atrophy study (PROSPECT-M-UK) with *FGF14* repeat expansion measure. The PROSPECT-M-UK study protocol was previously described elsewhere.^21^ Information on symptoms at onset and MSA clinical subtype was available for 138 MSA cases (83 male and 55 female) with non-expanded *FGF14*. Longitudinal data on disease milestones were available for 96 cases.

In addition, we genotyped the *FGF14* repeat locus in 1003 non-ataxic control individuals. The control cohort included 276 individuals of European origin from the Montreal Neurological Hospital, Montreal, QC, Canada, 202 individuals of European origin from the University of Tübingen, Tübingen, Germany, and 525 individuals of overwhelmingly European origin from the Children's Mercy Research Institute's Genomic Answers for Kids programme.

### DNA extraction

DNA was extracted from blood for clinically diagnosed MSA cases, whereas for pathologically confirmed cases DNA was extracted from brain tissue. Depending on availability, DNA from 30 mg of frozen cerebellar or frontal cortex was extracted using the QIAamp DNA Mini Kit (Qiagen) according to the protocol and was stored at −80°C until use. A subset of MSA patients (*n* = 21) had matched DNA extracted from both blood and brain tissue.

### Genetic screening for *FGF14* repeat expansions

The *FGF14* repeat locus was genotyped by long-range (LR)-PCR. Repeat sizes were measured by capillary electrophoresis or, when not possible, agarose gel electrophoresis, as described previously.^22^ Patients with an allele of ≥200 triplets underwent bidirectional repeat-primed PCRs (RP-PCRs) targeting the 5′-end and the 3′-end of the locus to ascertain the presence of a GAA•TTC repeat expansion and interruptions.^22^ RP-PCR products were analysed on an ABI 3730*xl* or ABI 3130*xl* DNA Analyzer using the GeneScan 1200 Liz Dye Size Standard. Results were analysed using the software GeneMapper. We measured the length of uninterrupted GAA•TTC repeats, excluding polymorphisms and interruptions. Only uninterrupted GAA•TTC-pure expansions of ≥250 repeats were included in downstream analysis.

**Figure 2:**
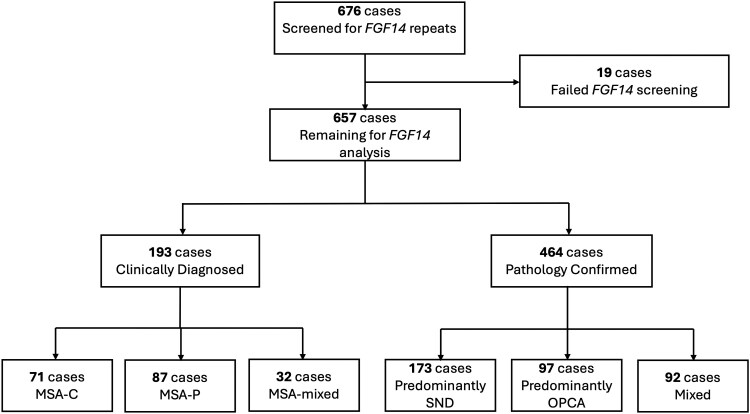
**Flowchart diagram of genetic tests performed in this study for confirmation and validation of *FGF14* GAA•TTC repeat expansions.** MSA-C = multiple system atrophy cerebellar subtype; MSA-P = multiple system atrophy parkinsonian subtype; OPCA = olivopontocerebellar atrophy; SND = striatonigral degeneration.

Alleles of ≥300 GAA•TTC repeats (GAA_>300_) were considered pathogenic, while alleles of 250–299 GAA•TTC repeats (GAA_250–299_) were considered likely to be pathogenic with incomplete penetrance, based on previously published data (Fig. 2).^12,13,15^

Sanger sequencing of PCR amplification products was performed in cases with an allele of ≥200 triplets at the Centre d’expertise et de services Génome Québec using the ABI3730*xl* DNA Analyzer (Applied Biosystems) and in the Laboratoire de Génétique du Centre Hospitalier Régional Universitaire de Nancy using the ABI3130*xl* DNA Analyzer (Applied Biosystems), as described previously.^22^

The control cohorts from Montreal and Tübingen were genotyped using a standardized PCR-based protocol,^22^ whereas the Children's Mercy Research Institute control cohort was genotyped by long-read PacBio HiFi sequencing, as reported previously.^23^

### Long-read sequencing of cases with *FGF14* GAA expansions

Cases with *FGF14* expansions of ≥250 repeats were analysed further with long-read sequencing using Oxford Nanopore Technologies (ONT). The LR-PCR amplicons were normalized to 150 ng/µl, then multiplexed using native barcoding expansion PCR-free library preparation kits and the SQK-LSK110 sequencing kit according to the manufacturer's instructions (Oxford Nanopore Technologies), multiplexed and sequenced on the PromethION platform using the R10.4.1 flow cell (Oxford Nanopore Technologies). Each run included a negative control. Reads were base-called and demultiplexed using Guppy. Sequences were aligned to the GRCh38 reference human genome.

The ‘Noise-Cancelling Repeat Finder’ (NCRF, v.1.01.02) was used to analyse the *FGF14* trinucleotide repeats^24^ from long-read sequencing data. To filter for the long allele, a threshold of 200 repeats was set, and only reads with a maximum noise of 80% were included in the analysis. The repeat length was determined with the median repeat length of all reads, as previously reported.^25^

### Statistical analysis

Categorical variables are reported as numbers and percentages. Continuous variables are presented as the mean ± standard deviation (and median/range for the expanded cases as small numbers). Groups were compared using Fisher's test for categorical variables and Kruskal–Wallis and ANOVA tests for continuous variables, with a two-tailed type I error of 0.05. We used Spearman rank correlations to assess the relationship variables. Bonferroni corrections were applied to adjust for multiple comparisons. All statistical analyses were carried out with STATA v.17.0 software.

## Results

### Demographic features of MSA subjects

We screened 657 MSA cases for the *FGF14* GAA•TTC repeat expansion, of which 464 were pathologically confirmed MSA and 193 were clinically diagnosed. Demographics of the study participants are summarized in Table 1. In the clinically diagnosed MSA cases, 45.8% (*n* = 87/190) had MSA-P, 37.4% (71/190) had MSA-C and 16.8% (*n* = 32/190) had MSA-mixed subtype. In the pathologically confirmed cohort, 45.3% (*n* = 150/331) had predominantly SND subtype, 26.9% (89/331) had predominantly OPCA subtype and 27.8% (*n* = 92/331) had a mixed phenotype (similar level of SND and OPCA involvement). Most cases (93.5% overall, of which 89% were clinically diagnosed and 95% pathologically confirmed MSA) were of European origin.

**Table 1:** Allelic frequency of *FGF14* GAA expansions in multiple system atrophy

Group	All MSA screened (*n* = 657, alleles = 1314)		Pathologically confirmed MSA (*n* = 464, alleles = 928)		Clinically diagnosed MSA (*n* = 193, alleles = 386)	
	GAA_≥300_	GAA_250–299_	GAA_≥300_	GAA_250–299_	GAA_≥300_	GAA_250–299_
MSA cases	7 (1.06%)	12 (1.83%)	5 (1.07%)	6 (1.29%)	2 (1.04%)	6 (3.11%)
Controls (*n* = 1003)	2 (0.19%)	12 (1.20%)	2 (0.19%)	12 (1.20%)	2 (0.19%)	12 (1.20%)
*P*-value	**0.033**	0.30	**0.036**	1	0.12	0.056

Statistical significance was set at *P* < 0.05 using Fisher's exact test. Statistically significant results are highlighted in bold. AF = allele frequency; MSA = multiple system atrophy.

There were no statistically significant differences in sex proportion and the MSA subtype (MSA-P/C or mixed) between the clinically diagnosed and the pathologically confirmed MSA cohorts. Information on age at onset, clinical subtype and survival was available in 521 of 657 cases and on pathology subtype (SND/OPCA/mixed) in 331 of 464 pathologically confirmed cases. A significantly younger age at onset (58.0 ± 9.5 versus 59.9 ± 8.6 years, *P* = 0.02) and shorter survival (7.6 ± 3.4 versus 8.2 ± 3.1 years, *P* = 0.02) were noted in the pathologically confirmed MSA cohort compared with the clinically diagnosed MSA group (Supplementary Table 1).

People with cerebellar-predominant phenotype had an earlier age of onset in both the clinical cohort (57.9 ± 8.6 years, adjusted *P* = 0.05) and the pathologically confirmed cohort (56.7 ± 8.7 years, adjusted *P* = 0.05) (Supplementary Table 2). The age of onset in the clinical cohort was 60.7 ± 8.7 years for MSA-P and 62.3 ± 7.8 years for mixed MSA. In the pathologically confirmed cohort, age of onset was 59.3 ± 9.7 years in SND cases and 56.5 ± 9.7 years in mixed cases. No differences in disease duration were noted between clinical subtypes in the clinically diagnosed or pathologically confirmed MSA cohorts.

### Distribution of *FGF14* GAA•TTC repeat expansion in MSA versus healthy controls

Using a combination of LR-PCR and bidirectional repeat-primed PCRs in the whole cohort (*n* = 657 MSA cases, 1314 chromosomes) we identified 19 MSA cases carrying an *FGF14* GAA_≥250_ expansion (2.89%, 19/657). Of these, 7 MSA cases (1.07%, 7/657) carried an *FGF14* GAA_≥300_ expansion (Supplementary Fig. 1) and 12 MSA cases (1.83%, 12/657) carried an *FGF14* GAA_250–299_ expansion (Table 1 and Supplementary Fig. 2). We found no cases carrying biallelic expansions. We also detected 10 non-GAA-pure expansions. RP-PCR accurately detected polymorphisms and interruptions within these expanded alleles, and the results were consistent with long-read ONT sequencing. The frequency of GAA_≥250_ expansions in the pathologically confirmed MSA cohort (*n* = 464, 928 chromosomes) was 2.37% (11/464). Specifically, *FGF14* GAA_≥300_ expansions were identified in five pathologically confirmed MSA cases, corresponding to a frequency of 1.08% (5/464). The frequency of GAA_250–299_ expansions in this group was 1.29% (6/464). In the clinically diagnosed cohort (*n* = 193, 386 chromosomes), the frequency of GAA_≥250_ expansions was 4.15% (8/193), including 1.04% (2/193) for GAA_≥300_ expansions and 3.11% (6/193) for GAA_250–300_ expansions. The frequency of GAA_250–299_ and GAA_≥300_ expansions in controls was 1.20% (12/1003) and 0.19% (2/1003), respectively. One of the controls carrying a GAA_≥300_ expansion was aged 55 years at the time of DNA sampling and did not exhibit ataxia, and age at sampling was not available for the second control. The frequency of GAA_≥250_ expansions was significantly higher in the combined MSA cohorts (2.89%, 19/657) compared with controls (1.40%, 14/1003) {odds ratio, 2.10 [95% confidence interval (CI), 0.99 to 4.57]; Fisher's exact test, *P* = 0.04}. A significantly higher frequency of GAA_≥250_ expansions was observed in the clinically diagnosed MSA cohort (4.15%, 8/193 versus 1.40%, 14/1003; odds ratio, 3.05 [95% CI, 1.09 to 7.92]; Fisher's exact test, *P* = 0.01) but not in the pathologically confirmed MSA cohort (2.37%, 11/464 versus 1.40%, 14/1003; odds ratio, 1.71 [95% CI, 0.70 to 4.10]; Fisher's exact test, *P* = 0.20). We then compared the frequency of GAA_≥300_ and GAA_250–299_ expansions in cases and controls. We found that the frequency of GAA_≥300_ expansions was significantly greater in pathologically confirmed MSA (1.08%, 5/464) compared with controls (0.20%, 2/1003) (odds ratio, 5.44 [95% CI, 0.89 to 57.32]; Fisher's exact test, *P* = 0.036), whereas it did not differ significantly in clinically diagnosed cases (1.04%, 2/193 versus 0.20%, 2/1003; odds ratio, 5.23 [95% CI, 0.38 to 72.71]; Fisher's exact test, *P* = 0.12). The frequency of GAA_250–299_ expansions was similar between controls and pathologically confirmed MSA cases (1.29%, 6/464 versus 1.20%, 12/1003; odds ratio, 1.08 [95% CI, 0.33 to 3.14]; Fisher's exact test, *P* = 1) and clinically diagnosed MSA cases (3.11%, 6/193 versus 1.20%, 12/1003; odds ratio, 2.65 [95% CI, 0.80 to 7.73]; Fisher's exact test, *P* = 0.056).

**Figure 3:**
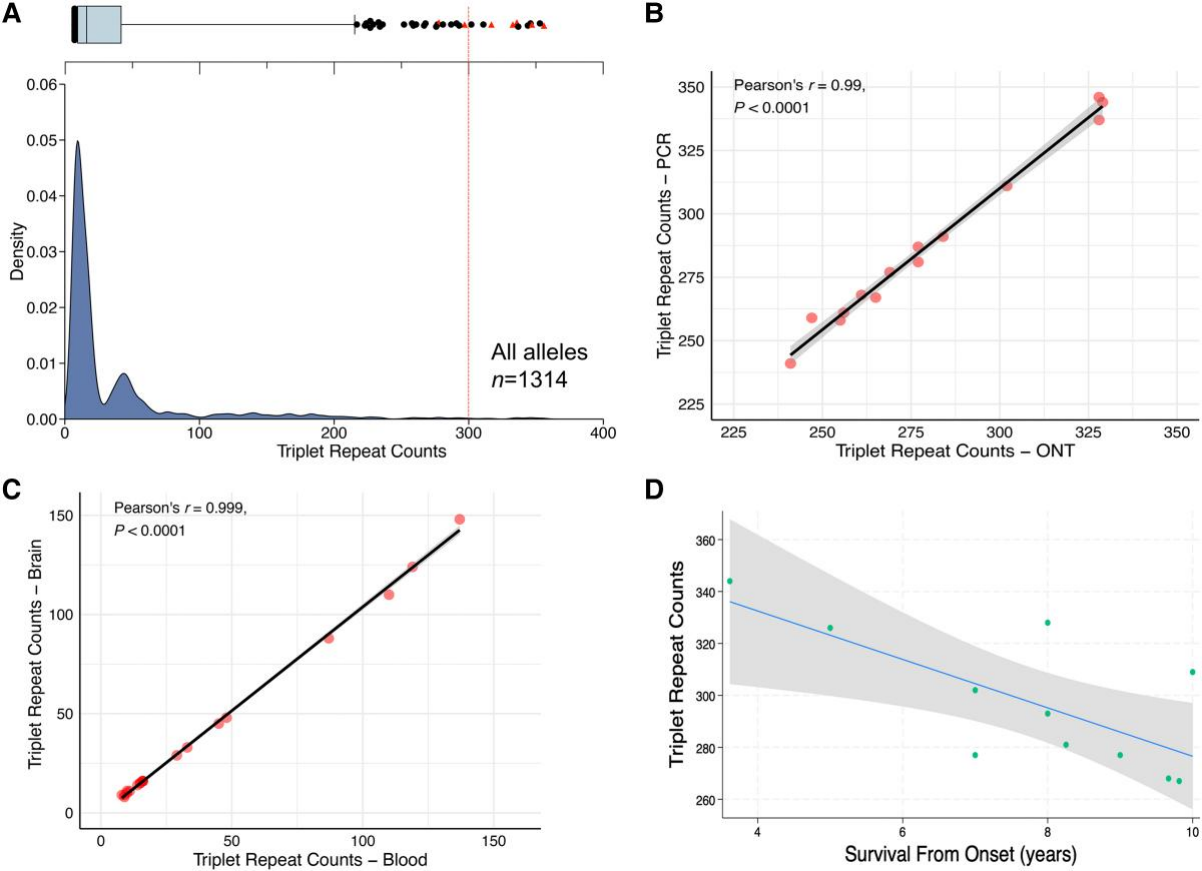
***FGF14* allelic distribution in MSA patients and correlation with survival.** (**A**) *FGF14* allelic distribution in all MSA patients included in this study. Allele distribution of the *FGF14* repeat locus in 657 MSA cases (1314 chromosomes). Among the patients with GAA-*FGF14*-positive MSA, seven were heterozygous for a GAA_≥300_ expansions, and 12 were heterozygous for a GAA_250–299_ expansion. The density plot shows allele-size frequencies, with higher densities indicating greater frequencies. The box-and-whisker plot shows the allelic distribution in patients. The box indicates the 25th percentile (first quartile), the median and the 75th percentile (third quartile), and the whiskers indicate the 2.5th and 97.5th percentiles. Outliers are represented by black dots. Expanded alleles consisting of non-GAA-pure repeats are represented by red triangles, and the red line marks the threshold of GAA_300_ repeat units. (**B**) Nanopore repeat size estimates concur with PCR estimates. Comparison of repeat size estimates by LR-PCR and ONT adaptive sequencing targeting for 14 individuals carrying a repeat expansion. (**C**) Correlation of repeat size in DNA from brain and blood matched samples. Correlation between size of the *FGF14* GAA•TTC repeat measured in matched samples from individuals with blood-extracted DNA and brain-extracted DNA (Pearson's *r* = 0.9, *P* < 0.0001). (**D**) Correlation between allele size and survival. Statistically significant negative correlation between size of the *FGF14* GAA•TTC repeat expansion and survival (calculated from disease onset until death) in patients with MSA (Pearson's *r* = −0.67; *P* = 0.02). LR-PCR = long-range PCR; ONT = Oxford Nanopore Technologies; MSA = multiple system atrophy.

We observed a wide variation of repeat sizes (Fig. 3A and Supplementary Fig. 3). In the pathologically confirmed MSA cases, the median size of GAA_≥300_ alleles and GAA_250–299_ alleles was 311 repeat units (range, 302–346) and 279 repeat units (range, 267–293), respectively. In the two clinically diagnosed cases, the sizes of the expanded GAA_≥300_ alleles were 337 and 353 repeat units, and the median size of the intermediate GAA_250–299_ alleles in the clinically diagnosed cases was 260 repeat units (range, 252–291). There was no statistically significant difference in repeat sizes in the two MSA diagnostic categories.

In our cohort, 12.9% of cases (85/657) had interruptions in the GAA repeats. Eight MSA cases (1.41%, 8/657), of which five were pathologically confirmed and two were clinically diagnosed, carried the (GAAGGA)*_n_* expansion (Supplementary Fig. 4). In comparison, two cases carried a different [(GAA)*_n_*(GCA)*_m_*]*_z_* expansion. These non-GAA-pure expansions were previously shown to be non-pathogenic conformation expansions for ataxia in individuals of European origin.^12,26,27^

### Long-read sequencing repeat expansion sizing concurs with PCR estimates

To assess further and to confirm the repeat expansions in *FGF14* GAA•TTC, we performed ONT sequencing in 25 cases. These included the cases with *FGF14* (GAA)_≥250_, cases with non-GAA-pure expansions and cases with complex GAA motif conformations detected by LR-PCR and RP-PCR. We found minimal difference in the number of GAA•TTC repeats between PCR-based and ONT estimates (Pearson's *r* = 0.99, *P* < 0.0001; Fig. 3B).

### Comparison between brain- and blood-extracted DNA

We obtained matched blood- and brain-derived DNA from 21 pathologically confirmed MSA cases, none of which carried an *FGF14* pathogenic repeat expansion. Data on brain tissue type was available in 14 of these cases; in nine cases DNA was extracted from cerebellum tissue and five cases from frontal cortex tissue. *FGF14* GAA•TTC allele sizes in matched frontal cortex tissue and blood-derived DNA were strongly correlated (Pearson's *r* = 0.9, *P* < 0.0001), irrespective of the repeat size of the largest allele (Fig. 3C). Two cases with GAA•TTC >100 repeats had a more than one repeat difference between blood and cerebellar brain tissue, GAA_137_ and GAA_119_ compared with GAA_148_ and GAA_124_ in the cerebellum (Supplementary Fig. 5). Alleles < 100 GAA•TTC repeats were of equal length in both brain and blood tissues.

### In-depth genotype–phenotype correlation of *FGF14* GAA_≥300_ patients with MSA

Seven patients diagnosed with MSA, of whom five (71.43%, 5/7) had pathological confirmation of diagnosis, carried a GAA_≥300_ expansion. In four of the five definite MSA cases, the neuropathological subtype was SND, whereas the other patient had mixed pathology. The two clinically diagnosed MSA patients with an *FGF14* GAA_≥300_ expansion had mixed MSA-P and C, and MSA-C predominant clinical phenotypes, respectively. The clinical characteristics of these patients are summarized in Supplementary Table 3 and the Supplementary material, ‘Data’ sections. The median age of onset in the *FGF14* GAA_≥300_ MSA cases was 58.6 (range, 49–69) years. We found no statistically significant correlation between onset age and the repeat expansion size (seven patients; Spearman's *r* = −0.3; *P* = 0.3). The first symptom experienced by patients at clinical onset was gait ataxia (50%, 3/6), parkinsonism (33.33%, 2/6) or autonomic failure (33.33%, 1/6), with data missing for one case (Table 2). None of the patients experienced episodic symptoms at disease onset (including episodic worsening of gait impairments or dizziness). The median disease duration from onset until death was 7.5 (range, 3.6–12) years in *FGF14* GAA_≥300_ cases, 8.3 (range, 4–11) years in *FGF14* GAA_250–299_ cases and 7.1 (range 3–12) years in *FGF14* GAA-negative MSA cases, with no statistically difference between the groups. At the time of MSA diagnosis, the cardinal clinical features in the seven *FGF14* GAA_≥300_ cases consisted of a combination of autonomic failure plus parkinsonism and autonomic failure plus cerebellar ataxia (data missing in one case). However, within the first year of symptom onset, five of six patients had progressed to exhibit the full triad of autonomic failure with parkinsonism and cerebellar ataxia.

**Table 2:** Clinical characteristics of MSA patients in relationship to their *FGF14* GAA repeat expansion status

Clinical characteristics	*FGF14* GAA_<250_	*FGF14* GAA_≥250_	*P*-value (adjusted *P*-value)	*FGF14* GAA_≥300_	*FGF14* GAA_250–299_	*P*-value (adjusted *P*-value)^a^
Diagnostic certainty for MSA						
Clinically diagnosed, % (*n*)	29 (185/638)	57.9 (11/19)	0.21(1)	28.6 (2/7)	50 (6/12)	0.63 (1)
Pathologically confirmed, % (*n*)	71 (453/638)	42.1 (8/19)	0.21(1)	71.4 (5/7)	50 (6/12)	0.63 (1)
MSA clinical subtype						
MSA-C, % (*n*)	32.1 (170/530)	27.8 (5/18)	0.70 (1)	14.3 (1/7)	36.4 (4/11)	0.59 (1)
MSA-P, % (*n*)	49.2 (261/530)	50 (9/18)	0.95 (1)	85.7 (6/7)	27.2 (3/11)	0.05 (0.75)
MSA-mixed, % (*n*)	18.7 (99/530)	22.2 (4/18)	0.75 (1)	0 (0/7)	36.4 (4/11)	0.11 (1)
MSA pathology subtype						
MSA-predominantly SND, % (*n*)	47.6 (168/353)	60 (6/10)	0.52 (1)	80 (4/5)	40 (2/5)	0.52 (1)
MSA-predominantly OPCA, % (*n*)	27.5 (97/353)	0 (0/10)	0.06 (0.96)	0 (0/5)	0 (0/5)	1 (1)
MSA-mixed, % (*n*)	24.9 (88/353)	40 (4/10)	0.28 (1)	20 (1/5)	60 (3/5)	0.52 (1)
First symptom at onset						
Gait ataxia, % (*n*)	31.9 (43/135)	35.3 (6/17)	0.78 (1)	40 (2/5)	33.3 (4/12)	1 (1)
Parkinsonism, % (*n*)	34.8 (47/135)	41.2 (7/17)	0.61(1)	40 (2/5)	41.7 (5/12)	1 (1)
Erectile disfunction (in males), % (*n*)	11.1 (9/81)	25 (2/8)	0.26 (1)	33.3 (1/3)	20 (1/5)	1 (1)
Neurogenic bladder, % (*n*)	11.1 (15/135)	23.5 (4/17)	0.23 (1)	0 (0/5)	33.3 (4/12)	0.26 (1)
Orthostatic hypotension, % (*n*)	13.3 (18/135)	0 (0/17)	0.22 (1)	0 (0/5)	0 (0/12)	N/A

Statistical significance was set at *P* < 0.05. MSA = multiple system atrophy; MSA-C = multiple system atrophy cerebellar subtype; MSA-P = multiple system atrophy parkinsonian subtype; *n* = number of cases with available information; N/A = not available; OPCA = olivopontocerebellar atrophy; SND = striatonigral degeneration.

^a^ *P*-values after adjusting for multiple comparisons are in parentheses.

Detailed clinical description from onset until death or last seen alive was available in six of the seven cases. Cerebellar features predominantly included gait ataxia (100%, 6/6), limb ataxia (66.7%, 4/6), dysarthria (83.3%, 5/6) with onset in the first 2 years of disease, dysphagia (66.7%, 4/6) and cerebellar ocular motor signs (33.3%, 2/6). No information was available specifically on downbeat nystagmus, diplopia, oscillopsia or vertigo in any of these cases. Non-cerebellar characteristics were consistent with MSA phenotype and included autonomic failure (100% of cases) consisting of neurogenic bladder dysfunction (100%, 6/6), orthostatic hypotension (66.7%, 4/6), erectile dysfunction (100% of male cases, 3/3), gastrointestinal symptoms (83.3%, 5/6) and sialorrhoea or sweating abnormality (50%, 3/6). Extrapyramidal features included bradykinesia (66.7%, 4/6) and mild rest tremor (33.3%, 3/6). Levodopa treatment was initiated in 83.3% (5/6) of patients, and minimal or no response was reported in all cases. Additional features included postural instability with retropulsion, polyminimyoclonus, cervical dystonia and depression. Despite the advanced age of the patients, cognitive impairment (based on clinical evaluation) was uncommon (16.6%, 1/6 patients). Sensation was normal in all patients. Disease progression was rapid in most cases. Walking aids were used in the first 5 years from onset (60%, 3/5), progressing to use of a wheelchair shortly after that in three of the five patients for whom data were available.

The median age at death was 63 years (range, 55–77 years), with no statistically significant correlation between age at death and the repeat expansion length (six patients; Spearman's *r* = −0.48; *P* = 0.2).

### In-depth genotype–phenotype correlation of *FGF14* GAA_250–299_ patients with MSA

Twelve patients diagnosed with MSA carried an *FGF14* GAA_250–299_ expansion, half of whom (6/12) had a pathologically confirmed diagnosis (Supplementary Table 3 and the Supplementary material, ‘Data’ sections). Most of these cases were of European descent (10/12, 83.33%), and none of them had episodic symptoms at disease onset. The *FGF14* GAA_250–299_ MSA cases were phenotypically similar to the *FGF14* GAA_≥300_ cases (Table 3 and Supplementary Table 3). Half of the patients had a predominant MSA-C phenotype (6/12, 50%), four patients had an MSA-P predominant phenotype (33.3%,4/12), and two patients had a mixed MSA phenotype (16.7%, 2/12). In the clinically diagnosed MSA cases (*n* = 6), five had an MSA-C predominant phenotype. Age of onset in the *FGF14* GAA_250–299_ MSA cases was on average 55.7 (±7.7) years. There was no correlation between age at onset and the size of the repeat expansion in patients with GAA_250–299_ (*n* = 12, Pearson's *r* = −0.3; *P* = 0.3).

**Table 3:** Clinical features, progression and *FGF14* GAA repeat expansion status in multiple system atrophy

Clinical characteristics	*FGF14* GAA_<250_	*FGF14* GAA_≥250_	*P*-value (adjusted *P*-value)	*FGF14* GAA_≥300_	*FGF14* GAA_250–299_	*P*-value (adjusted *P*-value)
Age of onset, years	58.7 (37.4–80 ± 9.3)	56.8 (42–73 ± 8.1)	0.38 (1)	58.6 (49–70 ± 9.0)	55.7 (42–73 ± 7.7)	0.47 (1)
Age of death, years	66.1 [60–72]	63 [59–71.4]	0.46 (1)	65.6 [8–75]	63.0 [60–69.3]	0.98 (1)
Disease duration, years	7.1 [5.1–9.5]	8 [7–9.7]	0.47 (1)	7.5 [5–10]	8.3 [7–9.7]	0.72 (1)
**Cardinal clinical features when MSA diagnosis was first suspected, % (*n*)**						
Parkinsonism	27.1 (26/96)	11.7 (2/17)	0.23 (1)	0 (0/6)	9.1 (1/11)	1.00 (1)
Parkinsonism and cerebellar ataxia	1.04 (1/96)	11. 8 (2/17)	0.05 (0.7)	0 (0/6)	18.2 (2/11)	0.52 (1)
Parkinsonism and autonomic failure	15.6 (15/96)	32.5 (4/17)	0.48 (1)	16.7 (1/6)	9.1 (1/11)	1.00 (1)
Cerebellar ataxia and autonomic failure	21.9 (21/96)	0 (0/17)	**0.04 (0.06)**	0 (0/6)	0 (0/11)	N/A
Parkinsonism, cerebellar ataxia and autonomic failure	9.4 (9/96)	11.8 (2/17)	0.67 (1)	16.7 (1/6)	9.1 (1/11)	1.00 (1)
**Clinical features at the time of last examination**						
Cerebellar syndrome, % (*n*)						
Gait ataxia	98.8 (83/84)	100 (17/17)	1.00 (1)	100 (6/6)	100 (11/11)	N/A
Limb ataxia (upper or lower)	98.8 (83/84)	68.8 (11/16)	**0.000 (0.001)**	66.7 (4/6)	70 (7/10)	1.00 (1)
Dysarthria	94.1 (80/85)	93.3 (14/15)	1.00 (1)	83.3 (5/6)	100 (9/9)	0.40 (1)
Dysphagia	71.4 (60/84)	81.3 (13/16)	0.55 (1)	66.7 (4/6)	90 (9/10)	0.51 (1)
Cerebellar ocular motor signs	84.7 (72/85)	73.3 (11/15)	0.28 (1)	33.3 (2/6)	100 (9/9)	**0.01 (0.028)**
Postural tremor	85.7 (72/84)	43.8 (7/16)	**0.001 (0.01)**	40 (2/5)	45.5 (5/11)	1.00 (1)
Autonomic features, % (*n*)						
Autonomic dysfunction (any)	98.8 (84/85)	100 (17/17)	1.00 (1)	100 (6/6)	100 (11/11)	N/A
Neurogenic bladder	95.3 (81/85)	100 (17/17)	1.00 (1)	100 (6/6)	100 (11/11)	N/A
Gastrointestinal features	83.5 (71/85)	80 (12/15)	0.72 (1)	100 (5/5)	70 (7/10)	0.50 (1)
Orthostatic hypotension	71.8 (61/85)	81.3 (13/16)	0.55 (1)	66.7 (4/6)	90 (9/10)	0.51 (1)
Erectile dysfunction in males	95.4 (41/43)	100 (8/8)	1.00 (1)	100 (3/3)	100 (5/5)	N/A
Parkinsonian syndrome, % (*n*)						
Bradykinesia	95.2 (80/84)	87.5 (14/16)	0.25 (1)	80 (4/5)	90.9 (10/11)	1.00 (1)
Postural instability	88.1 (74/84)	64.3 (9/14)	**0.03 (0.047)**	20 (1/5)	88.9 (8/9)	0.36 (1)
Rest tremor	50 (42/84)	30 (3/10)	0.32 (1)	60 (3/5)	0 (0/5)	0.16 (1)
Levodopa trial	75.5 (114/151)	77.8 (14/18)	1.00 (1)	83.3 (5/6)	75 (9/12)	1.00 (1)
Positive levodopa response	7.1 (8/113)	7.7 (1/13)	1.00 (1)	0 (0/6)	11.1 (1/9)	1.00 (1)
Minimal/partial levodopa response	50.4 (57/113)	30.8 (4/13)	0.12 (1)	33.3 (2/6)	22.2 (2/9)	1.00 (1)
Negative levodopa response	42.5 (48/113)	61.5 (8/13)	0.19 (1)	33.3 (2/6)	77.7 (7/9)	0.31(1)
Progression						
Falls in the first year from onset, % (*n*)	35.7 (20/56)	66.7 (10/15)	**0.03 (0.047)**	50 (2/4)	72.7 (8/11)	0.24 (1)
Disease duration to first falls, years	2.71 [0.52–3.88] (*n* = 27)	0.5 [0.5–3] (*n* = 14)	**0.01 (0.028)**	1.0 [0–1] (*n* = 3)	0.5 [0.5–3] (*n* = 11)	1.00 (1)
Use of walking aid in the first 5 years from onset, %, (*n*)	77.4 (41/53)	76.9 (10/13)	1.00 (1)	75 (3/4)	77.7 (7/9)	0.47 (1)
Disease duration to regular use of walking aid, years	3.4 [2.1–4.8] (*n* = 34)	3.45 [1.0–6.0] (*n* = 11)	0.2 (1)	5 [2–7] (*n* = 3)	3.5 [0.75–5.5] (*n* = 8)	0.54 (1)
Regular use of wheelchair in the first 5 years from onset, % (*n*)	60.5 (23/38)	68.75 (11/16)	0.336 (1)	40 (2/5)	100 (8/8)	0.04 (0.06)
Disease duration to regular use of wheelchair, years	7 [3.4–7] (*n* = 15)	5 [4.5–6] (*n* = 8)	**0.02 (0.039)**	5.5 [4–7] (*n* = 2)	5 [5–5] (*n* = 6)	0.787 (1)

Values are presented as the mean (range ± standard deviation), median [interquartile range] or percentage (*n*). Statistically significant values at *P* < 0.05 after adjusting for multiple comparisons are shown in bold. *n* = number of cases with available information; N/A = not available.

The first symptom experienced by patients at clinical onset was gait ataxia (33.3%, 4/12), autonomic failure (25%, 3/12), parkinsonism with autonomic failure (25%, 3/12) or parkinsonism without autonomic failure (16.7%, 2/12). Dysphagia and dysarthria were frequent features in this subgroup, with 83.3% (5/6) of patients experiencing them in the first 5 years from onset. More than half of the patients had upper limb ataxia and dysdiadochokinesia (58.3%, 7/12). Autonomic dysfunction was present in all patients, either as the first symptom (66.6%, 8/12) or soon after the disease onset. It comprised orthostatic hypotension (90%, 9/10), neurogenic bladder (100%, 11/11) and erectile dysfunction (100%, 5/5 male patients). Other symptoms experienced and signs noted in this group included rapid eye movement sleep behaviour disorder (77.7%, 7/9), stridor (44.4%, 5/9), broken pursuit (66.6%, 6/9) and horizontal gaze-evoked nystagmus (22.2%, 2/9). Ten patients were treated with levodopa. Apart from three who reported mild clinical benefits, all the others did not experience any benefit from levodopa therapy. Sensation was normal in all patients, and cognitive function remained preserved, even in advanced disease stages. Apart from Case C1, all clinically diagnosed patients had died by the time of this analysis, and the median disease survival in this group was 8.3 years (range, 4–11 years). Postural instability with retropulsion was more frequent in MSA cases without the *FGF14* expansion (adjusted *P* = 0.047).

### Impact of the *FGF14* expansion on the MSA phenotype

We compared three groups defined by the size of the longest allele: <250 repeats (*n* = 638); 250–299 repeats (*n* = 12); and ≥300 repeats (*n* = 7) (Table 2). There was no statistically significant difference in sex distribution, age at onset, predominant symptoms at onset or disease duration between the three groups (Table 2).

A subgroup of the MSA cases was recruited prospectively into a natural history study (*n* = 96). We assessed the clinical progression and disease milestones in MSA patients with *FGF14* GAA_≥250_ and in those with *FGF14* GAA_<250_. Importantly, none of the *FGF14* GAA_≥250_ cases presented with cerebellar ataxia with autonomic failure alone (in the absence of parkinsonism) compared with the non-expanded group (21.9%) (*P* = 0.04, adjusted *P* = 0.06). Furthermore, a higher proportion of cases with dual pathology compared with *FGF14*-negative MSA cases presented with a mixed phenotype of parkinsonism and cerebellar ataxia (11.8%) compared with MSA patients without the *FGF14* expansion (1.04%) (*P* = 0.05, adjusted *P* = 0.06). There were no significant differences in the age of onset, disease duration or symptom of onset between the two groups. In the 15 *FGF14* GAA_≥250_ MSA patients with information on the time of onset of falls, 10 (66.6%) experienced frequent falls within the first year, a significantly higher proportion than that of the *FGF14* GAA_<250_ MSA cases (37.7%, 20/56) (*P* = 0.03, adjusted *P* = 0.047). The median time from onset to falls was significantly shorter in *FGF14* GAA_≥250_ MSA (0.5 years, *n* = 14) compared with the *FGF14* GAA_<250_ MSA cases (2.71 years, *n* = 27) (*P* = 0.01, adjusted *P* = 0.028) (Table 3). Likewise, a shorter time to regular wheelchair use was observed in MSA patients with *FGF14* expansions (median 5 years, range 2–8 years) compared with MSA cases without *FGF14* expansions (median 7 years, range 4–11 years) (*P* = 0.02, adjusted *P* = 0.039). At the time of the last examination, a statistically significant higher number of patients presented with moderate or severe dysphagia in the MSA cases with *FGF14*-GAA_≥250_ (81.3%) compared with MSA patients without the *FGF14* expansion (74.1%) (*P* < 0.001).

The frequency of the *FGF14* GAA_≥250_ repeat expansion was 2.2% (8/249) among patients with MSA-P, 2.4% (4/166) among patients with MSA-C, and 4.8% (5/104) among patients with MSA-mixed phenotype. The frequency was 4.7% (6/126) among patients with rapidly progressive disease (patients who died within 5 years from onset). None of the patients with disease onset before age 45 years carried an *FGF14* GAA•TTC repeat expansion. In the entire cohort, an earlier age of onset was associated with a shorter survival (Pearson's *r* = −0.39, *P* < 0.001). Furthermore, in the pathologically confirmed MSA cases carrying an *FGF14* GAA_≥250_ repeat expansion, we found a negative correlation between the *FGF14* GAA•TTC repeat expansion size and disease survival (*n* = 11, Pearson's *r* = −0.67, *P* = 0.02) (Fig. 3D and Supplementary Table 4).

### Neuroimaging features

**Figure 4:**
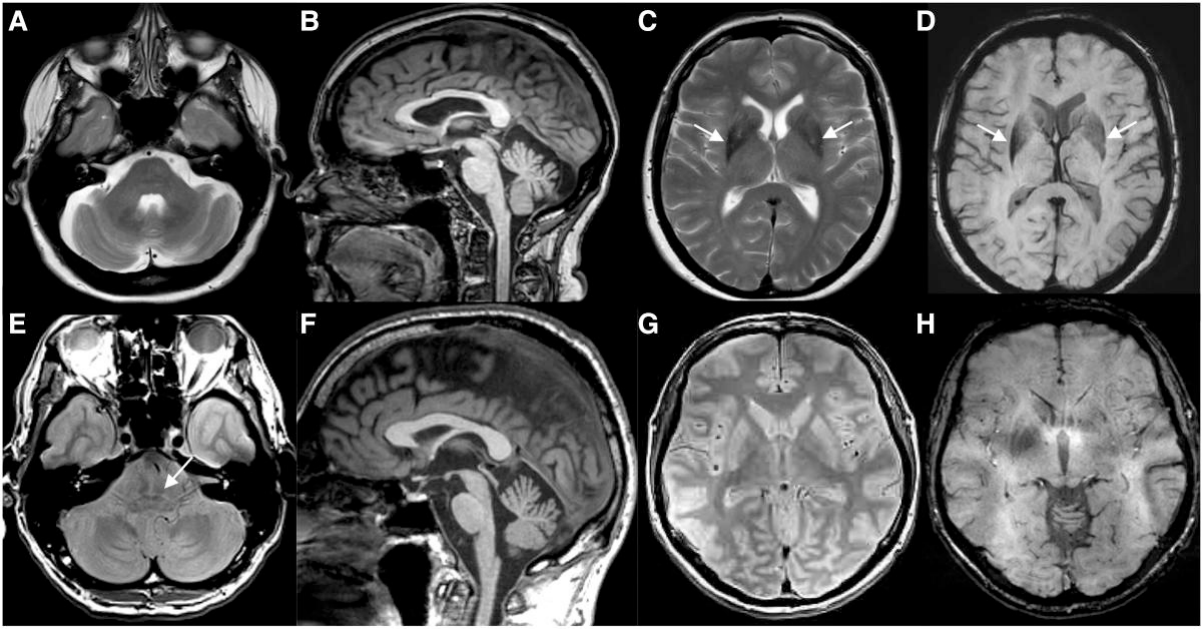
**MRI features in *FGF14* GAA_≥300_ patients with MSA.**  *Top panels* show MRI features in case C1 at age 59, 1 year from onset of symptoms, and *bottom panels* show MRI features in P3 at age 55, 5 years from onset of symptoms. (**A** and **C**) Axial T2-weighted images. (**B** and **F**) Sagittal T1-weighted images. (**E** and **G**) axial proton-density images. (**D** and **H**) Axial susceptibility-weighted images. Case C1 had a clinically established diagnosis of multiple system atrophy parkinsonian subtype and *FGF14* GAA_353_ repeat expansion. Case P3 had a neuropathologically established diagnosis of mixed type of multiple system atrophy and *FGF14* GAA_326_ repeat expansion. ‘Hot-cross-bun’ signs (**A** and **E**) and cerebellar atrophy (**B** and **F**) are present in both cases, more pronounced in Case P3. Hypointensity of the putamen in Case C1 is seen in is seen in **C** and **D**.

Brain MRI was available for two cases carrying an *FGF14* GAA_≥300_ expansion (Fig. 4) and nine cases carrying an *FGF14* GAA_250–299_ expansion (Supplementary Table 3). All 11 cases had MRI findings consistent with an MSA diagnosis. Atrophy of cerebellar hemispheres and/or vermis assessed on visual inspection was reported in six (54.5%), brainstem atrophy in five (45.4%) and putamen atrophy in three (27.2%). Hyperintensity of the middle cerebellar peduncle was reported in three (27.2%), and a ‘hot-cross-bun sign’ sign in two patients (18.1%). None of the cases had a disproportionate cerebellar atrophy at the time of the MRI reports. Two patients had a dopamine active transporter (DAT) scan, both showing bilaterally reduced uptake in the basal ganglia.

### Neuropathological examination of MSA cases with *FGF14* (GAA)_≥250_

**Figure 5:**
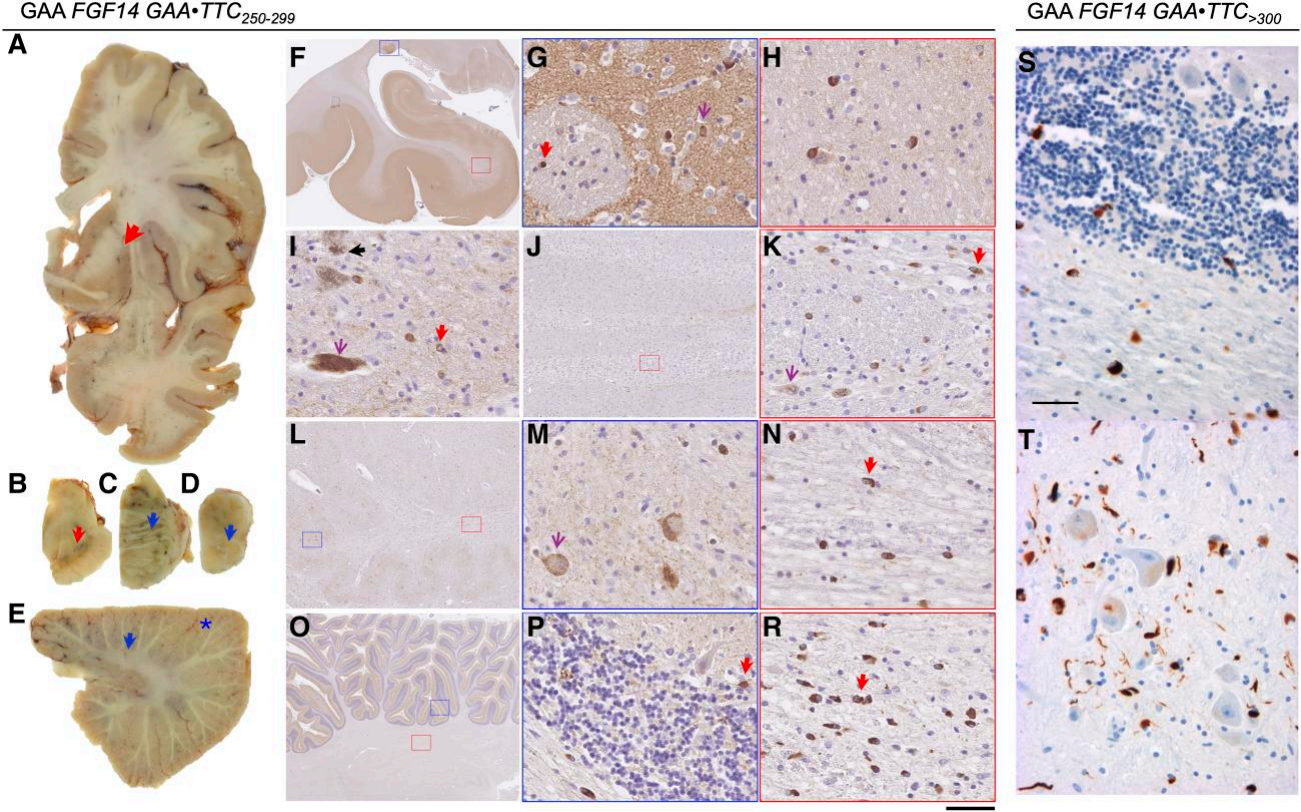
**Pathological findings in MSA cases with *FGF14* GAA repeat expansions.** Findings in *FGF14* GAA_≥300_ and *FGF14* GAA_250–299_ patients with MSA. (**A**–**E**) Case P7 (281 GAA repeats). (**A**) Severe putaminal atrophy, typical of MSA, is seen on the coronal section (red arrow). (**B**) Substantia nigra in the midbrain shows prominent pallor (red arrow). (**C**) The height of the pontine base is preserved (blue arrow). (**D**) The inferior olivary nucleus is clearly visible (blue arrow). (**E**) In the cerebellum at the level of the dentate nucleus, there is no evidence of significant white matter atrophy (blue arrow), the cerebellar cortex shows no apparent atrophy (blue asterisk), and the dentate nucleus is unremarkable. The macroscopic appearances are typical of MSA-SND. (**F**–**R**) Case P10 (277 GAA repeats). (**F**) Hippocampus shows no atrophy. (**G**) In the tail of the caudate nucleus (blue rectangle in **F**), there are glial cytoplasmic inclusions in the grey matter and within striato-pallidal fibres (red arrow), in addition to neuronal cytoplasmic inclusions in the grey matter (magenta arrow). (**H**) In the white matter of the parahippocampal gyrus (red rectangle in **F**) and adjacent gyri, there are occasional glial cytoplasmic inclusions. (**I**) In the substantia nigra, there is prominent depletion of pigmented neurons, with neuromelanin deposition freely in the neuropil (black arrow); occasional residual pigmented neurons contain diffuse cytoplasmic α-synuclein aggregates, and there are occasional glial cytoplasmic inclusions across the midbrain (red arrow). (**J**) In the pontine base, there is a prominent atrophy of the transverse fibres and pontine base nuclei (red rectangle in **J**). (**K**) There are numerous neuronal cytoplasmic and intranuclear inclusions (magenta arrow) in the pontine nuclei, and glial cytoplasmic inclusions (red arrow) in the nuclei and transverse fibres. (**L**–**N**) In the medulla, there are frequent diffuse neuronal cytoplasmic inclusions in the inferior olivary nucleus (blue rectangle in **L** and magenta arrow in **M**) and glial cytoplasmic inclusions in the white matter (red rectangle in **L** and red arrow in **N**). In the cerebellum (**O**, **P** and **R**), there is a moderate depletion of Purkinje cells, but good preservation of the granule cells, with occasional glial cytoplasmic inclusions (red arrow in **P**) in the cortex (magenta rectangle in **O**), and numerous glial cytoplasmic inclusions (red arrow in **R**) in the cerebellar white matter (magenta rectangle in **O**). The histological appearances are typical of MSA with equal SND and OPCA involvement. (**S** and **T**) α-Synuclein immunohistochemistry in findings in *FGF14* GAA_≥300_-positive patients with MSA show numerous intra-oligodendroglial inclusions in the cerebellum (**S**) and medulla oblongata (**T**). Scale bars: 3 mm in **F** and **O**; 50 µm in **G**–**I**, **K**, **M**, **N**, **P** and **R**; 400 µm in **J**; 0.7 mm in L; 40 µm in **S** and **T**. MSA = multiple system atrophy; MSA-C = multiple system atrophy cerebellar subtype; MSA-P = multiple system atrophy parkinsonian subtype; OPCA = olivopontocerebellar atrophy; SND = striatonigral degeneration.

Two cases with *FGF14* (GAA)_≥300_ and five cases with *FGF14* (GAA)_250–299_ had the neuropathology examination repeated after the *FGF14* expansion was identified (Fig. 5). The two cases with *FGF14* (GAA)_≥300_ were Patient P3 and Patient P4 (Supplementary material, ‘Data’ sections). The neuropathological features observed in these seven cases fell within the expected spectrum of MSA pathology. Macroscopic examination in Patient P4 performed at 8 years from disease onset (Fig. 5) showed a severe atrophy of the cerebellum predominating in the superior vermis, dentate nucleus and middle cerebellar peduncles. The inferior olivary nucleus was macroscopically normal. The pyramidal tracts appeared brownish. The pallidum, thalamus and sub-thalamic nuclei were normal, in contrast to the severe atrophy of the putamen. Microscopically, the putamen, substantia nigra and pontine nuclei showed a massive neuronal depopulation, with astrocytic gliosis. Abundant oligodendroglial cytoplasmic inclusions positive for α-synuclein were found in the striatum, pons, cerebellum, bulbar olive, transverse and perpendicular pontine fibres. The cerebellum showed a moderate loss of Purkinje cells, with numerous empty baskets and torpedoes. The glomeruli in the granular layer were rarefied. The cerebellar white matter was atrophic and contained numerous α-synuclein-positive oligodendroglial inclusions.

Neuropathological examination at 5 years from onset in Patient P3 showed no macroscopic abnormalities, in particular no cerebellar atrophy, no pallor of the substantia nigra and no abnormal staining of the putamen. Microscopic examination of the cerebellum revealed no white matter atrophy or loss of Purkinje cells. The only abnormality was a pallor in the hilum of the dentate nucleus. In the putamen, there was no notable neuronal loss, gliosis or pigmentary deposits, but there were numerous rounded intra-oligodendroglial inclusions labelled with anti-α-synuclein and anti-ubiquitin antibodies. In the cortical areas, particularly in the frontal regions, there were numerous α-synuclein-positive inclusions in the grey and white matter. In the substantia nigra, there was neuronal loss but no Lewy bodies. α-Synuclein immunohistochemistry showed numerous oligodendroglial and neuronal inclusions, which were also associated with dystrophic neurites. α-Synuclein-labelled inclusions were also abundant in pontine nuclei, medulla oblongata and cerebellum. None of the areas examined showed polyglutamine inclusion (polyQ, 1C2 antibody), as is common in spinocerebellar ataxias.

In the three cases carrying an *FGF14* (GAA)_250–299_ expansion and olivopontocerebellar atrophy (Patients P6, P9 and P10), macroscopic and microscopic examination showed cerebellar white matter atrophy and moderate depletion of Purkinje cells, with good preservation of the granule cell layer and mild gliosis in the molecular layer. In the two MSA cases with striatonigral degeneration dominant pattern (Patients P7 and P8) there was no macroscopic evidence of cerebellar cortical or white matter atrophy in one case. Microscopically, however, glial cytoplasmic inclusions were evident in the cerebellar white matter (Fig. 5). The dentate nucleus showed no significant atrophy, and none of the cases with intermediate expansions showed an unusual cerebellar atrophy pattern, exceeding the extent expected to be present in MSA. The pathology observed in these cases was of a classic MSA pathology without any pathological evidence to suggest cerebellar cortical atrophy beyond what is typically seen in MSA. Quantitative measurements were not applicable, because there was no obvious additional cortical cerebellar atrophy.

## Discussion

In our study, we investigated the frequency and potential contribution of *FGF14* GAA•TTC repeat expansions to the MSA phenotype by screening a large cohort of both clinically diagnosed and pathologically confirmed MSA patients. We found seven MSA patients carrying an *FGF14* GAA_≥300_ repeat expansion, five of whom were pathologically confirmed as fulfilling the gold standard neuropathological diagnosis of MSA. All five cases with pathologically confirmed *FGF14* GAA_≥300_ had typical α-synuclein-positive glial cytoplasmic inclusions (GCIs) and no atypical features for MSA on autopsy. None of these five patients reported a family history of either MSA or other forms of ataxia. The two clinically diagnosed MSA cases with *FGF14* GAA_≥300_ had typical clinical and radiological features fulfilling both the second consensus statement on the diagnosis criteria^4^ and the retrospectively applied Movement Disorder Society (MDS) criteria for a MSA diagnosis^7^ and were followed up longitudinally. These findings underscore the importance of genetic screening in this patient population, because it may have diagnostic and clinical implications. The discovery of *FGF14* GAA•TTC repeat expansions in a subset of pathologically confirmed MSA patients in our study introduces significant challenges for accurate and complete clinical diagnosis of these two conditions (MSA and SCA27B), when they co-exist.

We found *FGF14* GAA_≥300_ expansions in 1.06% (7/657) of the total MSA cohort and GAA_250–299_ expansions in 1.83% (12/657) of the cohort. Two healthy controls (2/1003, one aged 55 years) also carried *FGF14* GAA_≥300_ expansions. Interestingly, most of the *FGF14* (GAA)_≥300_ expansions uncovered in this study were relatively small (maximum size was 353 GAA repeats) in comparison to expansions in SCA27B patients, which can reach longer sizes.

Including our study, a total of 1843 MSA patients have been tested for the GAA•TTC expansions in *FGF14* (Supplementary Table 5). The published reports predominantly screened blood-extracted DNA from clinically diagnosed MSA-C cases, and almost all used the 2008 second consensus statement on MSA diagnosis criteria.^4^ Our study is the first to analyse a pathologically confirmed MSA cohort. Unlike most MSA cohorts screened for *FGF14* GAA•TTC expansions, which included predominantly MSA-C cases, our cohort screened all MSA phenotypes. We found a similar proportion of *FGF14* expansions in both MSA-C and MSA-P, highlighting the importance of genetic testing in both these presentations when a clinical suspicion arises, irrespective of the predominant MSA clinical subtype.

In our cohort, patients with MSA carrying an *FGF14* GAA_≥250_ repeat expansion exhibited a combination of autonomic failure, cerebellar ataxia and extrapyramidal symptoms, aligning with the classical MSA phenotype. However, we show that the *FGF14* GAA_≥250_ has an impact on the functional status of MSA patients, leading to a significantly higher proportion of cases presenting with falls in the first year from onset in the *FGF14* GAA-positive MSA patients compared with *FGF14* GAA-negative MSA cases (*P* = 0.03), a significantly reduced time to falls (*P* = 0.03) and shorter time to wheelchair use (*P* = 0.02). In comparison, many SCA27B patients remain ambulant after 15 years of disease.^17^ Shared characteristics with SCA27B include adult-onset cerebellar ataxia and, occasionally, a variable combination of mild pyramidal features, neurogenic bladder, autonomic dysfunction and, sometimes, parkinsonism. However, unlike SCA27B, the majority of MSA patients carrying an *FGF14* GAA_≥250_ repeat expansion reported here experienced early and severe gait ataxia and significant autonomic dysfunction, including neurogenic bladder and orthostatic hypotension from the disease onset. Autonomic dysfunction may occur in the later stages of SCA27B.^17^ Indeed, bladder symptoms, an early and highly disabling MSA feature, are usually reported in fewer than half the SCA27B patients, specifically described as urinary urgency or frequency.^28^ With the introduction of new MSA diagnostic criteria,^7^ the bladder features for either clinically probable or established MSA should include unexplained urinary retention and/or urinary incontinence, which should help to differentiate the urinary profiles of the two conditions. Dysarthria is absent in a substantial proportion of patients with SCA27B, even after a long disease duration.^17^ In our cohort of *FGF14* GAA-positive MSA cases, dysarthria was present from early stages and was severe, frequently associated with dysphagia. Furthermore, downbeat nystagmus and oscillopsia are frequent in SCA27B.^12,29^ However, in our study, horizontal nystagmus and interrupted saccades were the only ocular motor signs reported in the *FGF14* GAA-positive MSA cases, most probably related to incomplete data reporting, because these features in most cases were reviewed post-mortem from clinical notes. The subsequent disease course and progression of the *FGF14* GAA-positive MSA cases were consistent with a predominantly fast-progressing MSA and different from that of SCA27B. All cases included in this study fulfilled either the gold-standard neuropathological diagnosis of MSA or the highest diagnostic certainty of clinical diagnosis of MSA. Furthermore, we have no evidence that *FGF14* GAA•TTC repeat expansions contribute directly to MSA pathology or act as a causative factor for MSA. Instead, they are likely to represent a co-occurring, distinct condition that might modulate the clinical course of MSA, potentially contributing to a shorter survival, as we have shown in our cohort. This interpretation is supported further by the neuropathological findings of seven reported SCA27B cases, none of which showed evidence of α-synuclein pathology or features consistent with MSA. The clinical diagnosis for patients with both MSA pathology and an *FGF14* expansion remained compatible with MSA diagnosis, because their presentation aligns more closely with the characteristic features of MSA rather than the typical phenotype of SCA27B. SCA27B is defined by a late-onset, slowly progressive and rather pure pancerebellar syndrome, often with episodic symptoms at onset and cerebellar ocular motor signs, including downbeat nystagmus. Notably, a few studies have shown that *FGF14* expansions in the incompletely penetrant range (250–299 GAA repeats) may co-occur with other pathogenic variants associated with hereditary ataxias.^14,15^ In those cases, the clinical phenotype usually reflected the co-occurring variant with earlier onset and a more severe or complex presentation that deviated from the classic SCA27B phenotype. Likewise, in patients with both MSA pathology and *FGF14* expansions, the phenotype was dominated by MSA, supporting MSA as the primary clinical diagnosis in these cases. However, future studies comparing MSA patients with other neurodegenerative conditions, such as Parkinson's disease and dementia with Lewy bodies, could help to explore whether *FGF14* expansions are uniquely associated with MSA or are part of a broader neurodegenerative spectrum.

Recently, cerebellar ataxia with neuropathy and vestibular areflexia syndrome linked to biallelic AAGGG expansions in *RFC1* has attracted attention as a differential diagnosis of MSA^30^ and of SCA27B^28^; however, no *RFC1* expansions have been identified in pathologically confirmed MSA cases.^31^ Despite phenotypic overlap between MSA, *RFC1-*related disease and *FGF14* GAA-related disease, certain features can help to differentiate these disorders. Chronic cough, a prevalent feature in *RFC1*-related disease,^32-34^ was uncommon in our cohort. Motor neuropathy is typically absent or minimal in *RFC1*-positive patients,^32,35^ whereas it may co-occur with sensory neuropathy in some SCA27B patients^28^ but was absent in our MSA cases. Our patients did not exhibit any vestibular abnormalities, unlike a large proportion of patients who present with *RFC1-*related disease.

Importantly, the size of the GAA•TTC repeat expansion was inversely correlated with survival in MSA patients. Future studies should include detailed assessment of the severity of cerebellar involvement and related complications, such as dysphagia, aspiration pneumonia and increased frequency of early falls, in MSA cases with *FGF14* expansions and their impact on survival.

Our findings suggest that *FGF14* expansions might modulate the disease course in MSA, highlighting the need for genetic testing in these patients. No correlation was found with age of onset. In SCA27B, the size of the GAA•TTC repeat expansion shows only a weak negative correlation with age at onset^12,16^ and has not been associated with disease severity or progression.^15,17^ Significant phenotypic variability among patients carrying expansions of similar sizes has been reported in SCA27B, suggesting that additional factors influence disease expressivity.^15-17^

The phenotypic similarities between MSA and SCA27B complicate the clinical diagnosis, especially in the early stages of the disease. This study supports the integration of genetic testing for *FGF14* expansions in the diagnostic work-up of MSA, particularly for patients with early-onset cerebellar ataxia, parkinsonism and autonomic dysfunction. This approach could improve diagnostic accuracy and facilitate more personalized management strategies for patients with MSA. Screening for *FGF14* expansions is important for patient stratification in MSA trials, because the presence of these expansions can impact progression and survival in MSA.

Several cohorts of MSA-C or sporadic late onset cerebellar ataxia including MSA-C have been screened for *FGF14* GAA•TTC repeat expansions.^18,36-38^ Only one study had previously identified *FGF14* GAA_≥300_ repeat expansions in two patients initially diagnosed clinically as possible MSA.^18^ Based on the genetic findings and re-evaluation of their phenotype, a final diagnosis of SCA27B was reached for these cases. In a Chinese cohort of 527 MSA-C cases, four patients were found to have an intermediate expansion ranging in size from 264 to 275 repeat units.^37^ Similar findings were reported in two European cohorts^18,38^ and two Japanese cohorts.^36,39^ No detailed clinical description of the cases with intermediate expansion was presented in the European cohort. Studies on East-Asian populations did not find high frequencies of *FGF14* GAA expansions, with only one case having intermediate expansion identified in two Japanese cohorts.^39^ The patient was classified as SCA27B upon re-evaluation. SCA27B patients progressed more slowly than probable MSA-C patients in all the reported cohorts. These previous studies highlight the importance of *FGF14* screening in rare cases of SCA27B presenting early on as an MSA mimic.

Unlike previous reports of *FGF14* screening in MSA, most of the cases in our study (70%) had a pathologically confirmed diagnosis of MSA. Neuropathological studies in SCA27B have demonstrated that neuronal loss is predominant in the cerebellar cortex, especially in the vermis in comparison to the hemispheres.^12,17^ The neuropathological findings in SCA27B, described in seven cases to date, are largely restricted to the cerebellum.^12,17,40^ All cases displayed cerebellar cortical atrophy, more pronounced in the vermis than the hemispheres, with widespread loss of Purkinje neurons (most severe in the anterior vermis), mild granule-cell loss, and gliosis of the molecular layer. ‘Empty baskets’ were observed in less chronically affected regions. No substantial atrophy was observed in the dentate nuclei or cerebellar peduncles. Importantly, none of the cases exhibited intranuclear or cytoplasmic p62-positive inclusions, polyglutamine immunoreactivity, or α-synuclein or tau pathology in the cerebellum. Additionally, the substantia nigra showed no evidence of atrophy or neuronal loss. Mild neuronal loss and gliosis were noted in the inferior olivary and vestibular nuclei in some cases. A recent study that examined the somatic instability of the *FGF14* GAA•TTC repeat in peripheral tissues matched with post-mortem brains and uncovered a tendency for the repeat to expand somatically in cerebellar tissue.^41^ In our study, all five pathologically confirmed MSA cases with *FGF14* GAA_>300_ expansions were tested on DNA extracted from the cerebellum, which might account for the higher frequency of positive cases identified in this MSA cohort. Most of our MSA cases with an *FGF14* expansion had a variable combination of striatonigral degeneration and olivopontocerebellar involvement. This aligns with the clinical presentation of these patients, who often exhibited a mix of parkinsonism and cerebellar ataxia. Our study did not find significant differences in α-synuclein pathology on neuropathological examination among MSA patients with or without *FGF14* GAA•TTC expansions, suggesting that these genetic mutations might contribute to the MSA phenotype through mechanisms independent of α-synuclein aggregation. Previous autopsy of SCA27B cases did not find significant α-synuclein pathology in the brain.^12,17^ Further research is needed to elucidate how *FGF14* GAA•TTC expansion influences neurodegenerative processes and their interaction with synucleinopathies. To explore whether *FGF14* expansions lead to unique neuropathological alterations, beyond classical MSA features, particularly in the cerebellum, future studies would require advanced quantitative and molecular techniques for further morphometric analyses.

This study has several limitations. First, given that the cohort is primarily pathologically based, the ascertainment of age of onset and clinical milestones relied on patient medical record reassessment. Second, our study cohorts were predominantly of European ancestry, which might limit the generalizability of the findings to other populations. The relatively small number of cases found to carry an *FGF14* GAA•TTC expansion limits the statistical power of some analyses and the ability to draw definitive conclusions about the phenotypic differences between subgroups. Longitudinal studies are needed to gain a better understanding of the natural history of MSA in patients with *FGF14* expansions and to evaluate the potential therapeutic implications of these genetic findings.

## Conclusion

In conclusion, this study highlights the potential clinical and genetic overlap between MSA and SCA27B. The presence of these expansions in a subset of MSA neuropathologically confirmed patients underscores the need for genetic testing in the diagnostic evaluation of patients with clinical suspicion of MSA or in MSA patients with atypical and fast-progressing trajectories. These findings have important implications for improving diagnostic accuracy, drug trials and understanding the molecular underpinnings of MSA, ultimately contributing to better clinical management and therapeutic development for this complex neurodegenerative disorder.

## Supplementary Material

awaf134_Supplementary_Data

## Data Availability

Individual deidentified data may be shared with any qualified investigator on reasonable request (a data transfer agreement may be required, to specify conditions of use required to protect anonymity and remain consistent with participant consent).
