# Supplementary material for: Intronic FGF14 GAA repeat expansions impact progression and survival in multiple system atrophy
Source: Brain. 2025 Apr 16;148(9):3252–65. doi: 10.1093/brain/awaf134 (PMC12404725; doi:10.1093/brain/awaf134)
Supplement: awaf134_Supplementary_Data [file awaf134_supplementary_data.pdf]

Supplementary

Supplementary tables

Table 1. Demographics and clinical characteristics of all cases included in this study.

	**Entire MSA Cohort (n=657)**	**Clinically Diagnosed MSA (n=193)**	**Pathologically confirmed MSA (n=464)**	***P* Value (clinical versus pathologically confirmed diagnosis)**
**Demographics**				
**Male,** % (n)	52.3 (343)	57.5 (111)	50.1 (233)	0.1
**Age at onset,** years, mean (± SD)	58.7 (± 9.3)	59.9 (± 8.6)	58.0 (± 9.5)	**0.02**
**Disease Duration to death/last seen alive (survival)** years, mean (± SD)	7.8 (± 3.3)	8.2 (± 3.1)	7.6 (± 3.4)	**0.02**
**Clinical Subtypes**				
**MSA-C**, % (n)	31.9 (175/548)	37.4 (71/190)	29.4 (104/358)	0.13
**MSA-P,** % (n)	49.3 (270/548)	45.8 (87/190)	51.1 (183/358)	
**MSA-mixed,** % (n)	18.8 (103/548)	16.8 (32/190)	19.8 (71/358)	
**Pathology subtype**				
**Predominantly SND,** % (n)	NA	NA	45.3 (150/331)	NA
**Predominantly OPCA,** % (n)	NA	NA	26.9 (89/331)	
**Mixed,** % (n)	NA	NA	27.8 (92/331)	

Table S1. Demographic characteristics of all MSA cases included in the study. Legend: MSA=multiple system atrophy, n=number, NA=not available, MSA-C = MSA cerebellar subtype, MSA-P= MSA parkinsonian subtype, SND=striatonigral degeneration, OPCA=olivopontocerebellar atrophy, SD=standard deviation, IQR – interquartile range. Statistical significance set at p<0.05.

Table 2. Clinical features based on predominant clinical and pathological subtype.

	Clinically Diagnosed Cohort (188 cases with data available)				Pathologically Confirmed Cohort (317 cases with data available)			
	MSA-C	MSA-P	MSA-Mixed	*p* value (adj. p value)	MSA-C	MSA-P	MSA-Mixed	*p* value (adj. p value)
Age of onset, years, mean (± SD)	57.9 (± 8.6)	60.7 (± 8.7)	62.3 (± 7.8)	**0.03 (0.05)**	56.7 (± 8.7)	59.3 (± 9.7)	56.5 (± 9.7)	**0.03 (0.05)**
Post-hoc analysis for age of onset								
MSA-C	-	**0.005**	1	-	-	**0.039**	1	-
MSA-Mixed	1	**0.26**	-	-	1	**0.004**	-	-
Disease Duration/Survival, years, mean (± SD)	8.4 (± 2.7)	8.2 (± 3.6)	7.8 (± 2.8)	0.68 (1)	7.7 (± 3.1)	7.5 (± 3.3)	8.1 (± 3.6)	0.38 (1)

Legend: MSA=multiple system atrophy, MSA-C = MSA cerebellar subtype, MSA-P= MSA parkinsonian subtype, SD=standard deviation. Statistical significance set at $p<0.05$ after adjusting for multiple comparisons are bolded. All other items had significant unadjusted p-values, which were no longer significant after adjustment for multiple comparisons.

Table 3. In-depth clinical characteristics of MSA cases with *FGF14* GAA$_{\geq 250}$ expansions.

	FGF14 Pathogenic Range (GAA≥300)							FGF14 Intermediate range (GAA250-299)											
**ID**	**C1**	**P1**	**P2**	**P3**	**P4**	**C2**	**P5**	**C3**	**C4**	**C5**	**P6**	**P7**	**P8**	**P9**	**P10**	**P11**	**C6**	**C7**	**C8**
**Diagnostic certainty of MSA**	Clinically established	Definite	Definite	Definite	Definite	Clinically established	Definite	Clinically established	Clinically established	Clinically established	Definite	Definite	Definite	Definite	Definite	Definite	Clinically established	Clinically established	Clinically established
***FGF14* GAA larger allele size**	353	344	311	326	346	337	309	252	276	261	287	281	268	267	277	293	291	259	258
**DNA origin**	Blood	Brain	Brain	Brain	Brain	Blood	Brain	Blood	Blood	Blood	Brain	Brain	Brain	Brain	Blood	Brain	Blood	Blood	Blood
**Family history of MSA or ataxia**	No	No	No	No	No	No	Unknown	No	Mother-essential tremor	No	Unknown	Unknown	Unknown	No	No	No	No	No	No
**Age of onset (years)**	60	68.5	69	50	50	63	49	49	42	73	58	54	62	59	50	55	55	52	60
**Age of death (years)**	NA	72	77	55	58	75	59	NA	50	82	65	62	71	69.3	59	63	60	63	64
**Age at last examination (years)**	64	Unknown	77	54	57	72	Unknown	51	49	82	Unknown	61	68	Unknown	56	61	60	63	63
**Survival (onset to death, years)**	NA	3.6	7	5	8	12	10	NA	8	9	7	8	9	10	9	8	5	11	4
**Predominant MSA clinical subtype**	MSA-P	MSA-P	MSA-P	MSA-P	MSA-P	MSA-C	MSA-P	MSA-C	MSA-C	MSA-C	MSA-mixed	MSA-P	MSA-mixed	MSA-P	MSA-C	MSA-P	MSA-P	MSA-C	MSA-C
**MSA pathology findings and MSA subtype**	Alive at 65	a-syn positive GCIs, SND	a-syn positive GCIs, SND	a-syn positive GCIs, SND=OPCA	a-syn positive GCIs, SND	N/A	a-syn positive GCIs, SND	Alive at 55	N/A	N/A	a-syn positive GCIs, SND=OPCA	a-syn positive GCIs, SND	a-syn positive GCIs, SND	a-syn positive GCIs, SND=OPCA	a-syn positive GCIs, SND=OPCA	a-syn positive GCIs, SND	N/A	N/A	N/A
**Main clinical features at onset**																			
Cerebellar syndrome	Yes	No	No	No	Unknown	Yes	Unknown	Yes	Yes	Yes	No	No	No	No	No	No	No	No	Yes

(including gait ataxia)																			
Parkinsonism	No	Yes	Yes	No	Unknown	No	Unknown	No	No	No	Yes	Yes	Yes	Yes	No	No	Yes	No	No
Autonomic failure (genitourinary dysfunction and OH)	No	Yes	No	Yes	Unknown	No	Unknown	No	No	No	No	Yes	Yes	No	Yes	Yes	Yes	Yes	No
**Cardinal clinical features at the time of MSA diagnosis**																			
Parkinsonism	Yes	Yes	Yes	Yes	Yes	No	Unknown	Yes	Yes	Yes	Unknown	Yes	Yes	Yes	Yes	Yes	Yes	Yes	No
Cerebellar syndrome	Yes	Yes	No	Yes	Yes	Yes	Unknown	Yes	Yes	Yes	Unknown	Yes	No	No	Yes	Yes	Yes	Yes	Yes
Autonomic failure	Yes	Yes	Yes	Yes	Yes	Yes	Unknown	No	Yes	No	Unknown	Yes	No	Yes	Yes	Yes	Yes	Yes	Yes
**Clinical features at last examination**																			
**Cerebellar syndrome**																			
Gait ataxia	Yes	Yes	Yes	Yes	Yes	Yes	Unknown	Yes	Yes	Yes	Yes	Yes	Yes	Unknown	Yes	Yes	Yes	Yes	Yes
Limb Ataxia (upper or lower)	Yes	Yes	No	No	Yes	Yes	Unknown	Yes	Yes	Yes	Unknown	No	Yes	Unknown	Yes	No	No	Yes	Yes
Dysarthria	Yes	Yes	Yes	No	Yes	Yes	Unknown	Yes	Yes	Yes	Unknown	No	Unknown	Yes	Yes	Yes	Yes	Yes	Yes
Dysphagia	Yes	Yes	Yes	No	Yes	No	Unknown	Yes	Yes	Yes	Unknown	Yes	Yes	Unknown	Yes	Yes	Yes	Yes	No
Cerebellar oculomotor signs	Yes. Broken saccades, gaze nystagmus	Yes. Broken saccades, hypometric gaze nystagmus	No	No	No	Yes	Unknown	Yes. Broken saccades	Yes. Broken saccades. gaze nystagmus	Yes. Broken saccades	Unknown	Yes. Gaze nystagmus	Yes. Broken saccades	Unknown	Yes. Broken saccades	Yes	Yes.Hypometric sacades	Unknown	Yes. Broken saccades
Kinetic tremor	Yes	Yes	No	No	No	Yes	Unknown	No	Yes	Yes	Unknown	No	Yes	Yes	Yes	No	No	No	No
**Autonomic failure**																			
Autonomic dysfunction (any)	Yes	Yes	Yes	Yes	Yes	Yes	Unknown	Yes	Yes	Yes	Unknown	Yes	Yes	Yes	Yes	Yes	Yes	Yes	Yes

Neurogenic bladder	Yes	Yes	Yes	Yes	Yes	Yes	Unknown	Yes	Yes	Yes	Unknown	Yes	Yes	Yes	Yes	Yes	Yes	Yes	Yes
Gastrointestinal features	Yes	Yes	Yes	Unknown	Yes	Yes	Unknown	Yes	Yes	Yes	Unknown	Yes	Unknown	Yes	Yes	No	Yes	No	No
Orthostatic hypotension	Yes	Yes	Yes	No	Yes	No	Unknown	Yes	Yes	Yes	Unknown	Yes	Yes	Unknown	Yes	Yes	No	Yes	Yes
Erectile dysfunction (in men)	NA	Unknown	Yes	Yes	NA	Yes	Unknown	NA	NA	Yes	NA	Yes	NA	Unknown	NA	Unknown	Yes	Yes	Yes
Other (sialorrhea, abnormal sweating)	Yes	Yes	Yes	No	Unknown	No	Unknown	Unknown	Yes	No	Unknown	Unknown	Unknown	Yes	Yes	Unknown	Unknown	Yes	No
**Parkinsonism**																			
Bradykinesia	Yes	Yes	Yes	Yes	Yes	No	Unknown	Yes	Yes	Yes	Unknown	Yes	Yes	Yes	Yes	Yes	Yes	Yes	No
Postural instability with retro-pulsion	No	Yes	Yes	No	Yes	No	Unknown	Yes	Yes	Yes	Unknown	No	Yes	Yes	Unknown	Yes	Yes	Yes	No
Rest tremor	No	Yes	Yes	Yes	Yes	No	Unknown	No	No	No	Unknown	No	No	No	No	No	No	No	No
**Neuro-psychiatric features**																			
Cognitive Impairment	No	Unknown	No	No	No	Yes	Unknown	No	No	No	Unknown	No	No	Unknown	No	*No*	No	No	No
Behavioural abnormalities	No	Unknown	No	Yes	Yes	No	Unknown	Yes	No	No	Unknown	Yes	No	Unknown	No	*No*	No	No	No
**Other clinical features**																			
Myoclonus	No	Unknown	No	Unknown	Yes	No	Unknown	No	Yes	No	Unknown	Yes	Unknown	Unknown	Yes	Yes	No	No	Yes
Dystonia	Yes	Unknown	No	No	No	No	Unknown	No	Yes	No	Unknown	Unknown	Unknown	Unknown	Unknown	No	No	Yes	No
REM sleep behavioural disorder	Yes	Yes	No	No	Unknown	Yes	Unknown	Yes	Yes	Yes	Unknown	Yes	Unknown	Unknown	Yes	No	Yes	Yes	No
Stridor	No	Unknown	No	No	Unknown	No	Unknown	Yes	Yes	No	Unknown	Yes	Unknown	Unknown	Yes	No	Yes	No	No
Pyramidal syndrome	No	Yes	No	Unknown	Unknown	No	Unknown	No	No	No	Unknown	No	Yes	Unknown	Yes	No	Yes	Yes	No

Sensation	Normal	Unknown	Normal	Unknown	Unknown	Normal	Unknown	Normal	Normal	Normal	Unknown	Unknown	Normal	Unknown	Normal	Unknown	Unknown	Normal	Normal
**Clinical response to Levodopa treatment (in patients with parkinsonism)**																			
No response	No	Yes	No	Yes	Unknown	Yes	Unknown	Yes	Yes	Yes	Unknown	Yes	No	No	Yes	Yes	Yes	No	Unknown
Minimal response	No	No	No	No	Unknown	No	Unknown	No	No	No	Unknown	No	No	Yes	No	No	No	No	Unknown
Partial response	Yes	No	Yes	No	Unknown	No	Unknown	No	No	No	Unknown	No	Yes	No	No	No	No	Yes	Unknown
**Disease progression**																			
Falls in the first year from onset	Yes	Unknown	No	No	Unknown	Yes	Unknown	Yes	No	Yes	Unknown	Yes	Yes	Yes	No	Yes	Yes	No	Yes
Disease duration at first falls (years)	1	Unknown	6	NA	NA	0 (onset with falls)	Unknown	1	3	0.5	NA	0.5	0.5	0.5	6	0.5	0 (onset with falls)	4	0.5
Use of walking aid in the first 5 years from onset	Yes	Yes	Unknown	No	Yes	Yes	Unknown	Yes	Yes	Yes	Unknown	No	Yes	Unknown	No	Unknown	Yes	Yes	Yes
Disease duration at use of walking aid (years)	2	Unknown	7	NA	NA	5	NA	3	4	0.5	NA	NA	Unknown n	NA	6	NA	1	5	0.5
Use of wheelchair in the first 5 years from onset	Yes	Yes	No	No	Yes	No	NA	No	Yes	Yes	Unknown	Yes	Yes	Unknown	No	Unknown	Yes	Yes	Yes
Disease duration at regular use of wheelchair (years)	4	4	8	NA	NA	NA	NA	6	5	5	NA	Unknown	Unknown	NA	7	NA	5	5	2
Disease duration at dysarthria onset (years)	2	In the first 5 years, exact date unknown	Mentioned at 8 years, exact date unknown	Unknown	In the first 5 years, exact date unknown	Mentioned at 8 years, exact date unknown	Unknown	2	1	2	Unknown	Unknown	Unknown	In the first 5 years, exact date unknown	Unknown	Unknown	NA	2	Unknown

Disease duration at dysphagia onset (years)	2	4	8	Unknown	In the first 5 years, exact date unknown	NA		NA	1	2	Unknown	Unknown	Unknown	In the first 5 years, exact date unknown	Unknown	Unknown	NA	6	Unknown
Time to unintelligible speech	No	Unknown	No	Unknown	Unknown	No	Unknown	No	No	No	Unknown	Unknown	Unknown	Unknown	8	Unknown	NA	7.5	Unknown
Time to gastrostomy recommendation	No	Unknown	No	Unknown	Unknown	No	Unknown	No	No	No	Unknown	Unknown	Unknown	8	8	Unknown	NA	6	Unknown
UMSARS progression	Baseline =30 12 months =45 24 months =69	Unknown	NA	NA	NA	NA	NA	NA	Baseline=43 , 12 months =65	NA	NA	NA	NA	NA	Baseline=30 12 months =53 24 months =62 36months-80	NA	NA	NA	NA
**MRI findings**																			
Atrophy of cerebellar vermis/hemispheres	Yes	Unknown	Unknown	Yes	Unknown	Unknown	Unknown	Yes	Yes	No	Unknown	No	No	Unknown	Yes	Unknown	No	No	Yes
Brainstem atrophy	Yes	Unknown	Unknown	Yes	Unknown	Unknown	Unknown	Yes	Yes	No	Unknown	No	No	Unknown	No	Unknown	No	No	Yes
Basal ganglia	Yes, bilateral putaminal atrophy	Unknown	Unknown	No	Unknown	Unknown	Unknown	No	No	No	Unknown	No	Yes. Putaminal atrophy	Unknown	No	Unknown	Yes. Putaminal atrophy	No	No
T2/FLAIR abnormalities	Mild in midbrain	Unknown	Unknown	Middle cerebellar peduncle. “Hot cross	Unknown	Unknown	Unknown	Middle cerebellar peduncle	No	No	Unknown	Unknown	No	Unknown	Unknown	Unknown	No	No	Middle cerebellar peduncle. “Hot cross

				bun" sign															bun" sign
Age at MRI	59	NA	NA	55	NA	NA	NA	49	43	80	Unknown	59	68	Unknown	56	Unknown	56	59	61
DAT scan	Reduced uptake bilaterally									Reduced uptake bilaterally									

Legend: MSA-multiple system atrophy, n=number, NA=not available, MSA-C = MSA cerebellar subtype, MSA-P= MSA parkinsonian subtype, syn=synuclein, GCIs= glial cytoplasmic inclusions, SND=striatonigral degeneration, OPCA=olivopontocerebellar atrophy.

Table 4. Correlation between *FGF14* GAA repeat allele size and clinical features in cases with MSA.

		**Whole MSA Cohort**		**GAA$_{(\geq 250)}$ Cohort**		**GAA$_{(250\text{-}299)}$ Cohort**		**GAA$_{(\geq 300)}$ Cohort**	
		Pearson's correlation	P value	Pearson's correlation	P value	Pearson's correlation	P value	Pearson's correlation	P value
**Age of onset**	Clinically Diagnosed	-0.02	0.7627	0.22	0.5974	-0.20	0.7026	-1.00	1.0000
	Pathologically Confirmed	-0.04	0.4063	0.03	0.9283	-0.43	0.3914	0.007	0.9911
**Age of death**	Clinically Diagnosed	0.01	0.8871	0.18	0.7303	-0.48	0.4100	-0.13	0.8415
	Pathologically Confirmed	-0.05	0.2708	-0.16	0.6323	-0.60	0.2088	n/a	n/a
**Survival (onset to death, years)**	Clinically Diagnosed	0.07	0.3353	0.50	0.2019	-0.21	0.6909	-1.00	1.0000
	Pathologically Confirmed	0.0008	0.9876	**-0.68**	**0.02222**	**-0.88**	**0.0206**	-0.51	0.3758

Table 5. All published MSA cohorts screened for *FGF14* GAA expansions.

	Ouyang et al., 2024	Matsushima et al., 2024	Ando et al., 2024	Satolli et al., 2024	Wirth et al., 2024a	This study
Number of MSA cases screened	527	411	101	87	60	657
MSA phenotype	MSA-C	MSA-P, MSA-C	MSA-C	MSA-C	MSA-C	MSA-P, MSA-C, MSA-mixed
Number of cases with >300 GAA repeats (n,%)	0	0	0	0	2, 3.3%	7, 1.07%
Number of cases with 250-299 GAA repeats (n, %)	4, 0.75%	0	1, 0.99%	3, 3.44%	1, 1.6%	19, 2.89%
FGF14 repeat sizes (range)	264-275 GAA repeats	NA	273	NA	345-390	252-353
MSA diagnostic criteria used	2008	2008	NA	2022	2008	2008
DNA origin	Blood	Blood	Blood	Blood	Blood	**Brain (464)** and blood (193)
MSA diagnostic certainty of cases with *FGF* expansion	2 possible, 2 probable MSA	NA	Clinical diagnosis, no further details	Clinical diagnosis, no further details	2 possible, 1 probable MSA-C	**Definite MSA=464** Clinical MSA=193
Main clinical findings in MSA cases with FGF14 vs MSA	Slower progression MSA when *FGF14* expansion	NA	Initially diagnosed clinically as MSA-C with episodic symptoms at onset, later classified as SCA27B.	NA	Initially diagnosed clinically as MSA-C later classified as SCA27B. Slower progression in *FGF14* cases compared to MSA-C cases.	**Reduced survival** when FGF14 expansion

Supplementary Figures

Figure 1. Confirmation of *FGF14* GAA repeat expansions in MSA patients. The allele size of the *FGF14* GAA_n repeat expansion was determined in all available affected individuals (n = 657) using fragment analysis of long-range-PCR (LR-PCR) products**.** Electropherograms of 5' and 3' RP-PCR for the (GAA) repeat expansion demonstrated an extended saw-toothed consistent with a GAA expansion. IGV snapshots of the GAA_n locus in *FGF14* sequenced with targeted long-read Oxford Nanopore Technologies (ONT) confirmed the expansions in five cases with DNA available for analysis.

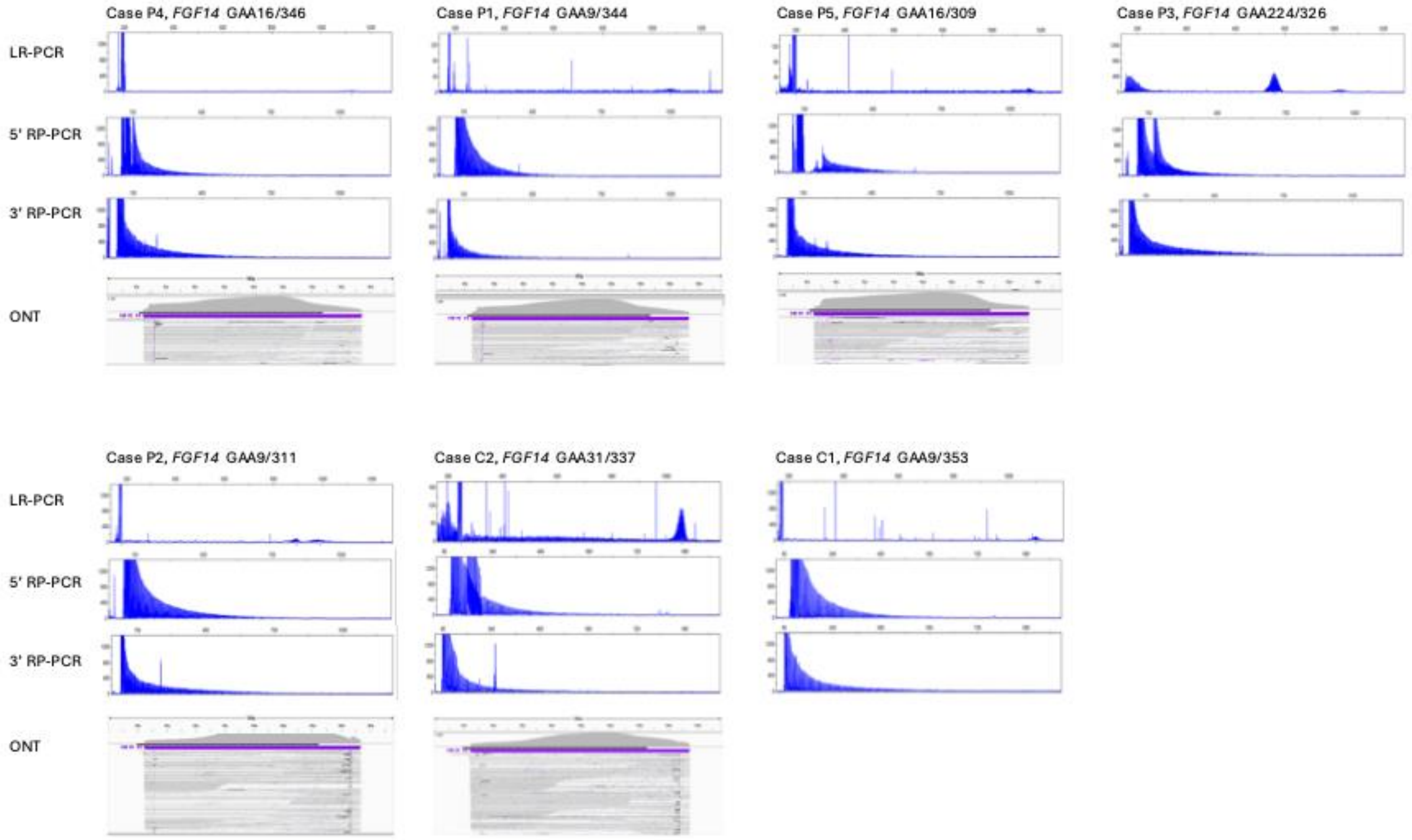

Figure 2. Confirmation of intermediate repeat expansions in *FGF14* in MSA patients. 12 individuals were found to have *FGF14* $GAA_{250-299}$ repeat using fragment analysis of long-range-PCR (LR-PCR) products**.** Electropherograms of non-expanded LR-PCR products displayed a single high intensity spike at <300 bp. Each case was further analysed with 5’ and 3’ RP-PCR for the (GAA) repeat expansion and demonstrated an extended saw-toothed product with three base pair repeat unit size in affected individuals.

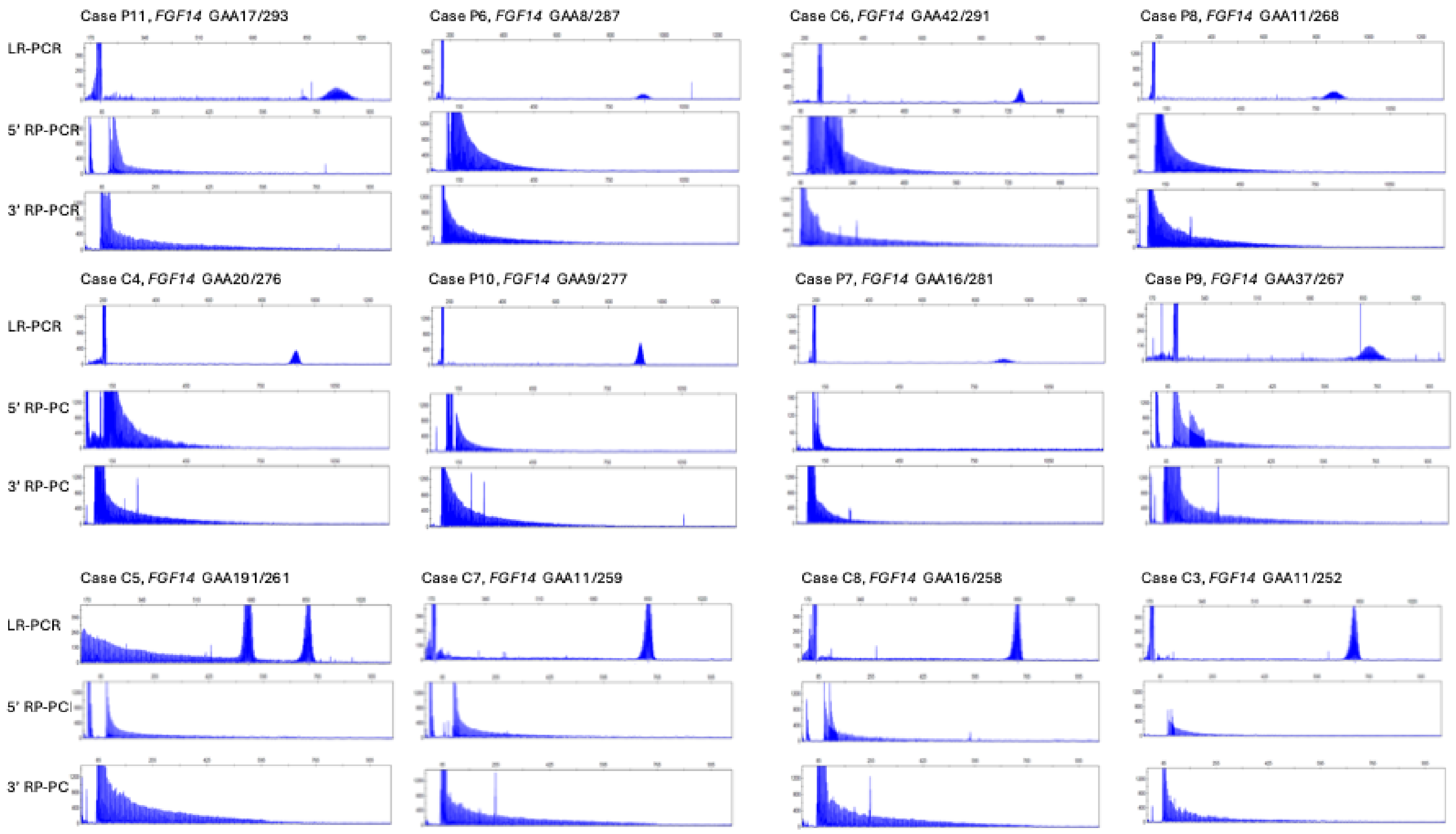

Figure 3. *FGF14* allelic distribution in MSA patients in the two diagnostic categories. Allele distribution of the *FGF14* repeat locus in clinically diagnosed and pathologically diagnosed MSA cases. The density plots show allele-size frequencies, with higher densities indicating greater frequencies. The box-and-whisker plots show the allelic distribution in patients. The box indicates the 25th percentile (first quartile), the median, and the 75th percentile (third quartile), and the whiskers indicate the 2.5th and 97.5th percentiles. Outliers are represented by black dots. Expanded alleles consisting of non-GAA repeats are represented by red triangles and the red line marks the threshold of GAA_{300} repeat units, above which the alleles are fully penetrant.

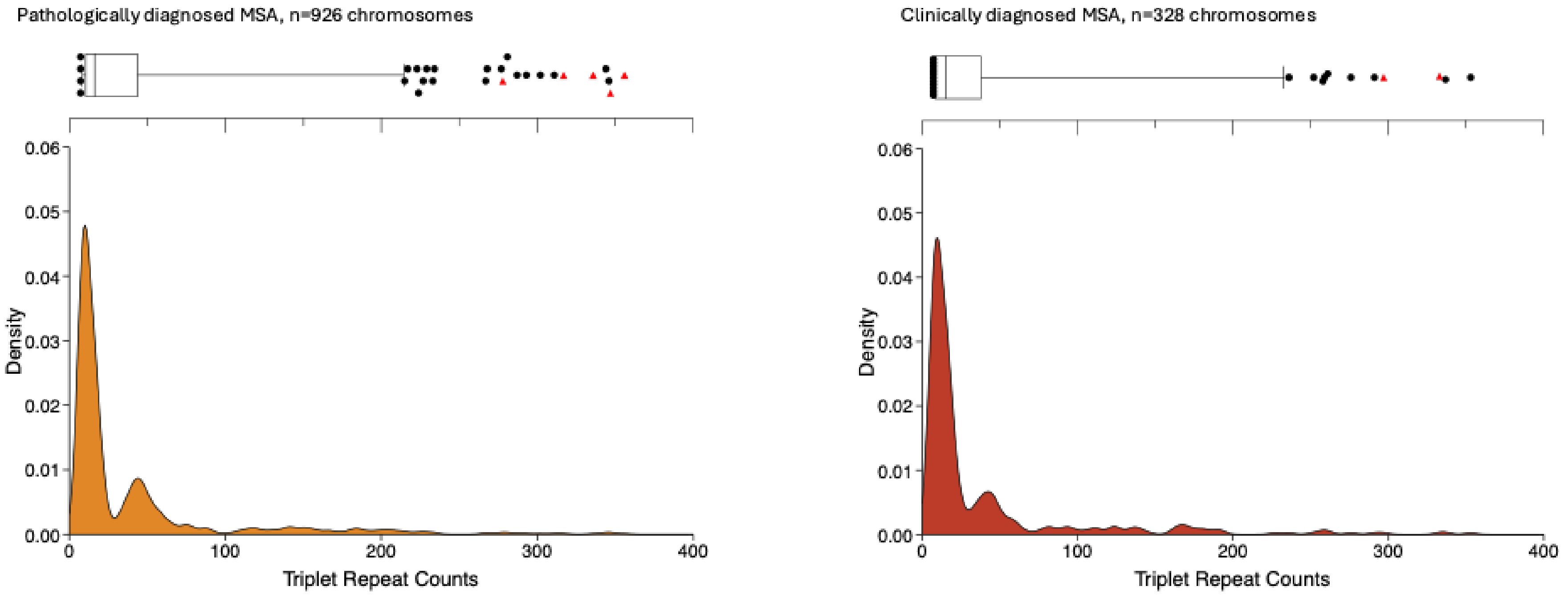

Figure 4. Confirmation of non-GAA repeat expansions in *FGF14* in MSA patients. RP-PCR analysis demonstrated inconsistent and stunted peak patterns, as opposed to the expected sawtooth “ladder.” This pattern is consistent with an impure/alternate repeat motif.

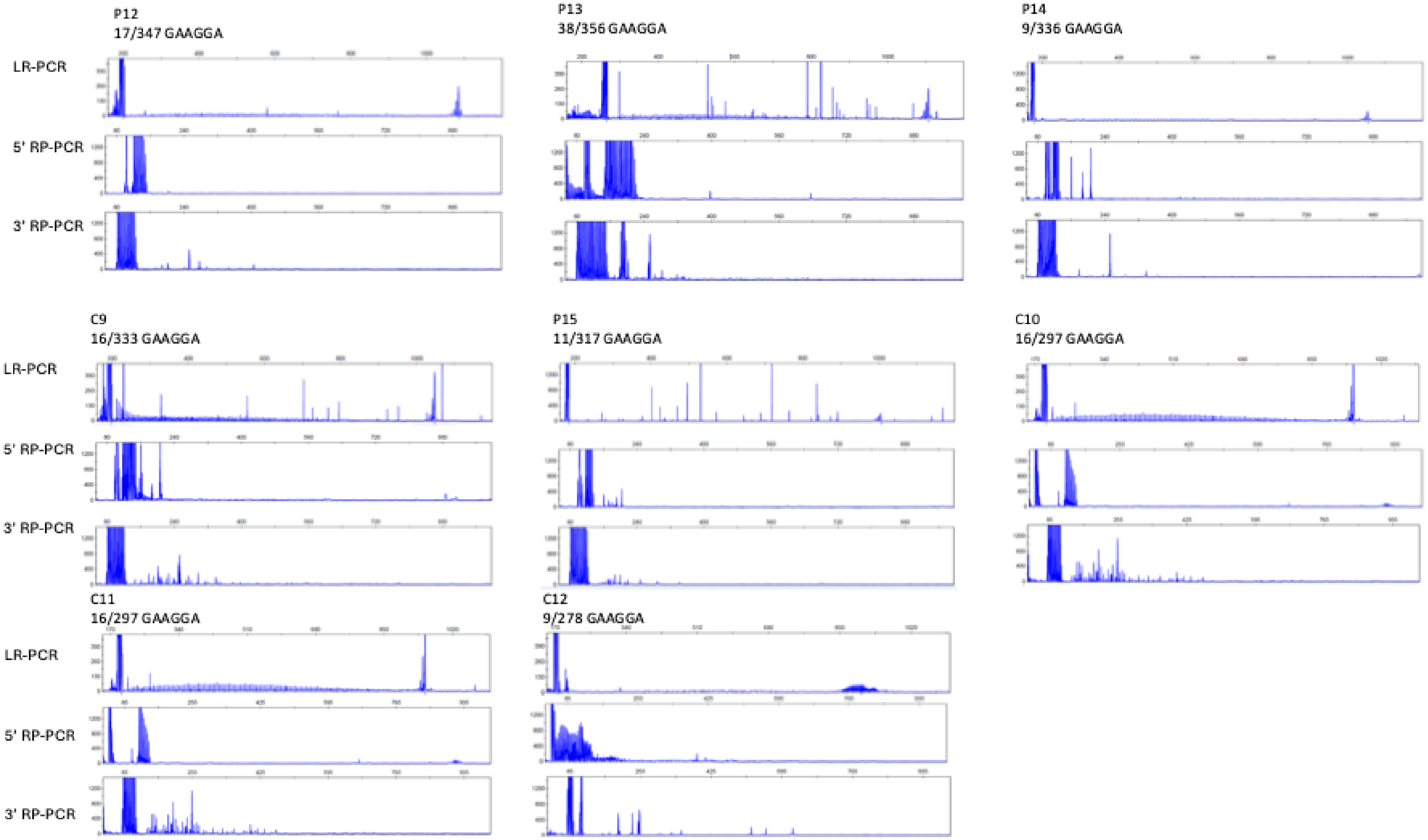

Figure 5. *FGF14* GAA•TTC repeat lengths correlation with blood in cerebellum and frontal cortex brain regions.

Profiles of the *FGF14* GAA•TTC repeat lengths, expressed in triplet repeat counts, across post-mortem brain regions and blood samples in three cases with pathologically established MSA. Observations for each of the two alleles from the same patient are connected by a dashed line.

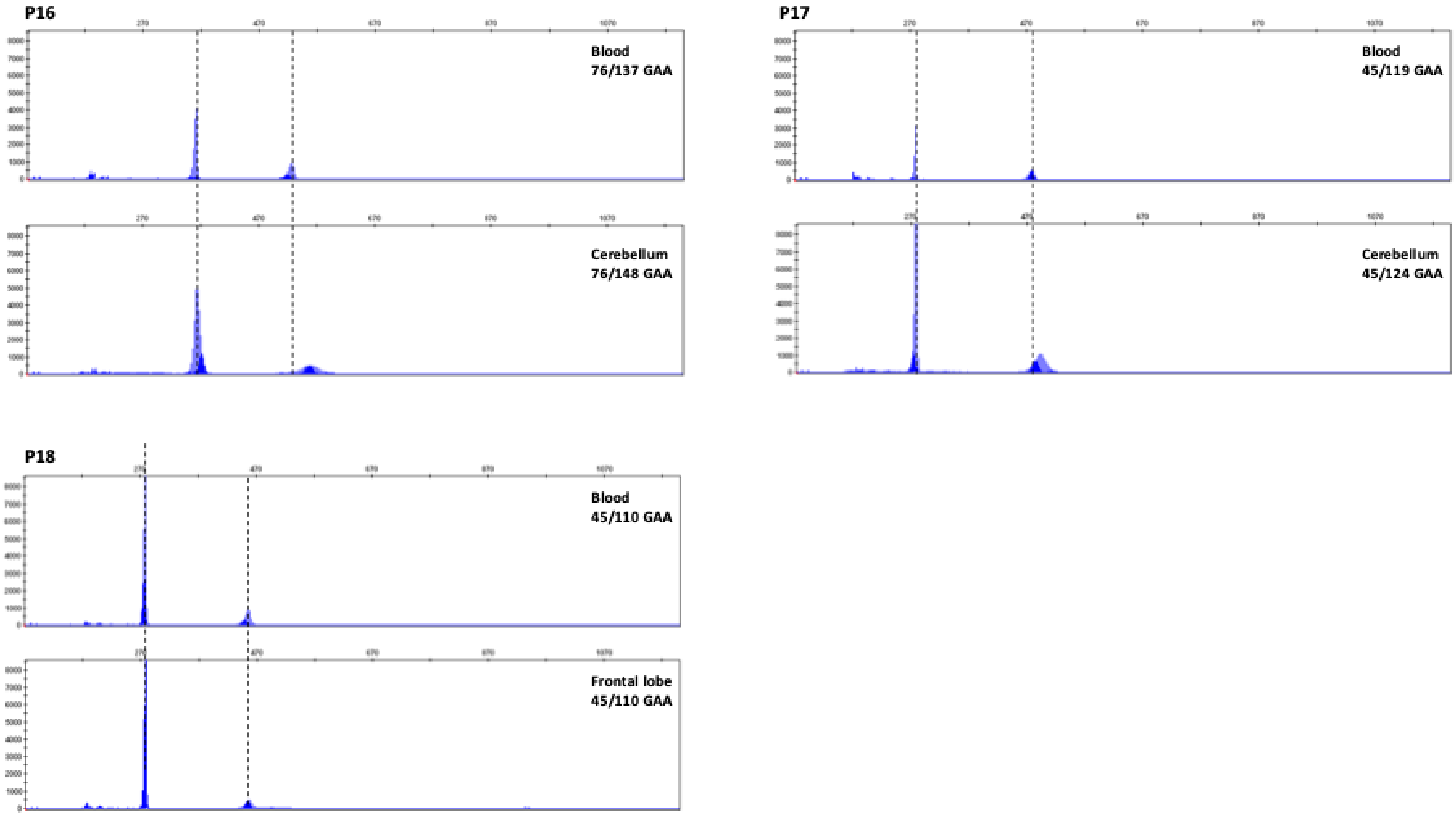

Supplementary data 1. Detailed clinical description of MSA cases with pathogenic *FGF14* expansions

Cases with *FGF14* fully penetrant GAA expansions

Case C1. Clinically established MSA, *FGF14* GAA_{353}

A 62-year-old female reported the occurrence of unsteady gait and frequent falls, generalised slowness when completing daily activities, postural light headedness, and intermittent left-hand tremor over a 2-year period. Past medical history comprised paroxysmal atrial fibrillation, ulcerative colitis, and irritable bowel syndrome. There was a family history (granddaughter) of leukodystrophy without a known underlying genetic cause, but no family history of any neurological conditions.

On examination, increased tone and bradykinesia (worse on the left) was noted. Gait was narrow-based and slow, and arm swing was significantly reduced. There was evidence of retropulsion and a tendency to fall backwards. Blood tests for vitamin B12 and serum folate, liver and kidney function including electrolytes, thyroid function, and full blood count were unremarkable. The brain MRI showed slightly more mineral depositions in the posterior putamina (more on the right than left) and mild cerebellar volume loss. Reduced radiotracer uptake in both basal ganglia (more marked on the right) was noted with presynaptic dopaminergic imaging. A diagnosis of clinically established MSA-P was made, and levodopa was started. She reported minimal subjective improvement in motor symptoms but worsening speech. Treatment was discontinued after 8 months.

Over the next 2-years she developed severe constipation and urinary hyperactivity, which was later managed with an indwelling catheter. Freezing of gait, cervical dystonia, and broken saccades with end-gaze nystagmus was also noted on examination. Her UMSARS total score at age 63 was 30, and it

progressed to 44 within 1-year and to 69 within 2-years (Video 1). She began using walking aids at age 62, and a wheelchair at age 64. Cognition remained intact and there were no reports of neuropsychiatric symptoms. Follow-up is ongoing.

Case P2, pathologically confirmed MSA, *FGF14* GAA_{311}

A 69-year-old male presented with rest tremor in the left arm, 'general slowness', erectile dysfunction, occasional night sweats, and 'jerk' episodes during sleep. Over the following two years the tremor evolved to include the right arm and legs. Past medical history included nephritis in childhood, hypertension, COPD and benign prostatic hypertrophy. There was no family history of note.

Examination at 71 revealed asymmetric rest tremor, mild bradykinesia, and reduced arm swing, all predominantly left-sided. Postural reflexes were impaired, walking was brisk and unaided, and he was able to turn, his eye movements were normal and denied orthostatic hypotension, micturition or bowel symptoms. The following year levodopa was commenced as tremor became bilateral. He had subjective improvement in mobility but no change in tremor. Over the next three years balance difficulties increased with occasional falls and urinary symptoms emerged. Re-examination at age 75 revealed bilateral rest tremor, mild cogwheel rigidity, and a stooped posture with reduced arm swing but with normal stride length and turn. Balance continued to deteriorate over the following year; levodopa was up titrated and pramipexole commenced.

On review at age 77, the falls had become frequent, urinary symptoms had progressed and he was experiencing postural light-headedness. He reported occasional difficulty swallowing dry food and started drooling at night; his speech was becoming slurred. At age 79 dysphagia had progressed to include fluids, a long-term urethral catheter had been inserted for managing progressing neurogenic bladder symptoms, had occasional faecal incontinence and he had become wheelchair dependent. The following year he sustained a neck of femur fracture falling from his wheelchair and died shortly after that.

Autopsy revealed mild enlargement of the ventricles but no other macroscopic cerebral pathology. The substantia nigra were mildly depigmented and the locus coeruleus was significantly atrophied. Macroscopic

cerebellar appearances were normal. Microscopic examination showed that the white matter of the cerebellum had some myelin pallor, and it was severely gliotic. A few Purkinje cells were atrophic. There was gliosis in the midbrain, with neuronal depletion and free-lying pigment in the substantia nigra. No obvious Lewy bodies were identified. Severe gliosis was also present in the pons and there was an area of tissue loss between the pontine nuclei. Gliosis was also seen in the sections from the medulla. Alpha-synuclein-positive glial cytoplasmic inclusions were seen in samples of the hippocampus, midbrain, pons and medulla, making the diagnosis of neuropathologically established MSA.

Case P1, pathologically confirmed MSA, *FGF14* GAA_{344}

70-year-old male presenting with poor balance, slowness and urinary incontinence, initially diagnosed with PD at the age of 68 years old. He was trialled on levodopa therapy but had no significant response. He was an ex-smoker with no significant past medical history. There was no family history of Parkinsonian or other neurological disorders.

Over the subsequent two years his symptoms progressed rapidly with deterioration in balance and mobility, worsening urinary incontinence, excessive sweating, the emergence of loud snoring at night, occasional choking while eating, drooling and slurred speech. On assessment aged 72, he was generally stiff with resting tremor in the right hand. Examination revealed hypomimia, increased tone in all four limbs and cogwheeling in the upper limbs which was more prominent on the right. There was wasting of the small muscles of the hand with fasciculations bilaterally. He was only able to stand for a short period and mobilise a few steps with support. Gait was festinant and balance was extremely poor, with inability to sit upright without support. Romberg's was untestable. On ocular examination pursuits were jerky, he had hypometric saccades both horizontally and vertically, and gaze-evoked nystagmus bilaterally. Coordination appeared mildy reduced and there was dysdiadochokinesia in the upper limbs.

Autonomic function tests at that time revealed sympathetic and parasympathetic failure with orthostatic and post-prandial hypotension. Video fluoroscopy revealed oropharyngeal dysphagia.

Shortly after, he was admitted to hospital with an aspiration pneumonia and died following respiratory arrest during the night, aged 72.

On postmortem the brainstem and cerebellum appeared macroscopically normal although the substantia nigra appeared pale. Microscopically there was moderate neuronal loss, gliosis and extracellular pigment deposition within the substantia nigra. Numerous glial cytoplasmic inclusions (GCIs) were seen in the substantia nigra and midbrain tegmentum with moderate numbers in the red nuclei. The locus coeruleus was depleted. There were no neurofibrillary tangles or Lewy Bodies within any of the pigmented nuclei or the cortex. There was Purkinje cell loss in the cerebellum with axonal swelling and gliosis. GCIs were present in the cerebellar white matter bundles. GCIs were also present in the putamen, thalamus, pons and inferior olive. The findings were consistent with a diagnosis MSA exhibiting more significant SND than OPCA pathology.

Case P3, pathologically confirmed MSA, *FGF14* GAA_{326}

The patients was examined at the age of 53 in the Neurology department of Pitié-Salpêtrière hospital for investigation of genito-urinary dysautonomia associated with gait problems. Past medical history comprised consisted of psoriasis, meniscectomy of left knee, arthritic discopathy, hemorrhoidectomy, cervico-prostatic incision. He had no family history of atypical Parkinsonism or other neurological conditions. He was previously treated for depressive symptoms with Paroxetine 20 mg.

He first experienced symptoms 3 years earlier with erectile dysfunction, for which he consulted an endocrinologist who prescribed intra-cavernous injections of alprostadil with no benefit. He then developed urinary urgencies and incontinence, for which he underwent a cervico-prostatic incision without any improvement. Dysphonia and problems with balance and gait had been progressively worsening for 1 year before his first examination at Pitié-Salpêtrière. He also complained about psychomotor slowing and fatigue.

On examination, he presented with a predominantly left akinetic-rigid syndrome, without resting tremor. He had left-sided action tremor. His gait was ataxic. He had no sensory, pyramidal, apraxic nor frontal symptoms. He had no orthostatic hypotension. Urodynamic evaluation showed a cervico-vescical

dyssynergy. Brain MRI was reported as normal. At that time a treatment with levodopa/carbidopa progressively increasing to 125 mg/3 time a day was started without clinical improvement. Six months later, the extrapyramidal and ataxic symptoms had worsened but without falls, whereas dysautonomia was stable; major depression with suicidal thoughts was reported. When the patient was included in the clinical trial BBIPS six month later, his motor symptoms had worsened and new symptoms such as insomnia and orthostatic hypotension were present. He died 6 months later, at the age of 55. Brain autopsy confirmed a definite MSA diagnosis.

Cases with *FGF14* intermediate range GAA expansions

Case C3, clinically established MSA, *FGF14* GAA_{252}

A 50-year-old female presented with an 18-month history of REM sleep behaviour disorder, poor balance, and frequent falls. She became more impulsive and experienced significant anxiety. Urinary frequency was reported and spanned several decades. There was no other past medical history to note, and family history was unremarkable.

Limb ataxia which was more marked in the lower limbs and a broad-based gait was noted on examination. No parkinsonian signs were noted. Blood tests for antinuclear antibodies, erythrocyte sedimentation rate, creatine kinase and Lyme disease were normal. Genetic testing for SCA1, 2, 3, 6, 7 and 17 expansions were negative. A brain MRI showed high signal changes within the middle cerebral peduncles bilaterally, with evidence of moderate cerebellar atrophy. Abnormal high signal pontine changes were noted within a “hot cross bun” configuration. Presynaptic dopaminergic imaging was normal.

Dysarthria developed at age 51, in addition to dysphagia. Alternating constipation and faecal incontinence also occurred. Autonomic profiling during this time showed evidence of cardiovascular failure, including mild orthostatic hypotension and abnormal blood pressure responses to the Valsalva manoeuvre. The working diagnosis was MSA-C at this stage. She progressed to using regular walking aids by age 53 and a wheelchair by age 55. She was intermittently using a urethral catheter from age 53, before receiving a

suprapubic catheter aged 55. Neuropsychiatric testing at age 54 and 56 demonstrated moderate cognitive decline with advancing anterior and subcortical dysfunction. Significant anxiety and depression developed in relation to personal circumstances from diagnosis and may have compounded cognitive testing. She continues to be reviewed in our service.

Case C4, clinically established MSA, *FGF14* GAA_{276}

A 46-year-old female presented with a 5-year history of balance difficulties, labile blood pressure with syncope, constipation, and urinary frequency, having initially been diagnosed with adult-onset ataxia. Falls occurred within 2-years of symptom onset. Past medical history was unremarkable. There was a family history of essential tremor (mother) and stroke (father), while parkinsonism without a specific underlying diagnosis was reported in both the maternal grandmother and paternal grandfather. None of these family members were available for further follow-up or DNA testing.

Extrapyramidal signs were noted on examination. In addition to this, dysmetria was observed in upper limbs, speech was hypophonic and gait was broad based. Blood tests for B12 folate, coeliac antibodies, cholestanol, alpha-fetoprotein, HIV and nicotinic ganglionic acetylcholine receptor antibodies were negative. Genetic testing for Friedreich's Ataxia and SCA1, 2, 3, 6, 7, 12 and 17 were also negative. Brain MRI showed mild-to-moderate cerebellar and brainstem atrophy, while presynaptic dopaminergic imaging was within normal limits. The diagnosis was revised to MSA-C at age 47.

She was re-examined at age 48 and found to have broken saccades and end-gaze horizontal nystagmus, cervical dystonia, and bilateral postural/kinetic tremor, in addition to the previously noted signs. Her UMSARS total score was 43, and this progressed to 65 within 12-months (Video 2). Levodopa was tried unsuccessfully during this period. The patient began using regular walking aids by age 47 and a wheelchair by age 49. They were intermittently self-catharizing (date of first use unknown) but began using an indwelling catheter by age 49. Cognition remained intact and there were no reports of neuropsychiatric symptoms. She passed away aged 50.

Supplementary data 2. The NNIPPS STUDY GROUP

Principal Investigator: P.N. Leigh (London, UK)
Co-ordination: European and UK: P.N. Leigh (London, UK), France: G. Bensimon (Paris, France), Germany: A.C. Ludolph (Ulm, Germany)
Steering Committee: Chair: P.N. Leigh (London, UK), Members: Y. Agid, G. Bensimon, M. Dib, L. Lacomblez, M. Vidailhet (Paris, France), D. Burn (Newcastle, UK); B. Landwehrmeyer, A.C. Ludolph (Ulm, Germany)
Independent Data Monitoring and Safety Committee: Chair: B. Asselain (Paris-France), Members: H. Allain (Rennes, France), D. Chadwick (Liverpool, UK), JE. Perret (Grenoble, France), C. Warlow (Glasgow, UK)
Technical Committees
Clinical diagnostic criteria: Chair: D. Burn (Newcastle, UK), Members: Y. Ben-Shlomo (Bristol, UK), AM. Bonnet, J. Fermanian, C. Payan, M. Verny, M. Vidailhet (Paris, France), P. Moore (Liverpool, UK), C. Tranchant (Strasbourg, France)
Motor Function, QoL & Health Service Research: Chair: C. Payan (Paris, France), Members: M. Borg (Nice, France), P. McCrone (London, UK), F. Durif (Clermont-Ferrand, France), A. Evans (London, UK), J. Fermanian (Paris, France), F. Viallet (Aix en Provence, France)
Neuro-Imaging: Chair: M. Verin (Rennes, France), Members: N. Deasy, J. Jarosz (London, UK), T.K. Hauser (Tübingen, Germany), E. Kraft (Ulm, Germany), E. Broussolle (Lyon, France), D. Dormont, C. Marsault, A. Tourbah (Paris, France), L. Defebvre, L. Delmaire (Lille, France), Y. Roland (Rennes, France)
Neuro-Pathology: Chair: J.J. Hauw (Paris, France), Members: C. Duyckaerts, D. Seilhean (Paris, France), S. Al-Sarraj, T. Revesz (London, UK), B. Landwehrmeyer (Ulm, Germany), H. A. Kretzschmar (Munich, Germany)
Neuropsychology: Chair: R. Brown (London, UK), Members: T. Bak (Cambridge, UK), A. Danek (Munich, Germany), B. Dubois, L. Lacomblez (Paris, France), RM. Marié (Caen, France), I. Uttner (Ulm, Germany)
Genetics: Chair: A. Dürr (Paris, France), Members: A. Al-Chalabi, N. Wood (London, UK), A. Brice (Paris, France), W. Camu (Montpellier, France), K. Morrison (Birmingham, UK)
Logistics, Treatments, Monitoring, Data Management & Statistical analysis
Chair: G. Bensimon (Paris, France), **European Project Manager**: M. Graf (Paris, France), **Data Manager**: C. Payan (Paris, France), **Data entry**: P. Paillasseur (Theriamis – St Maur des Fossés, France), **Senior Statistician**: C. Payan (Paris, France), Assistant Statistician: H.P. Pham (Paris, France), Functional scales development: J. Fermanian (Paris, France), Neuropsychology: R. Brown (London, UK), Health economics: P. Mc Crone (London, UK), **Clinical Research Assistants**: N. Dedise, C. Hermine, S. Sagnes, B. Poître, C. Foucart (Paris, France), A. Dougherty, C. Murphy, H. Mason (London, UK), T. Hermann, K. Klempp, A. Niess, V. Stange (Ulm, Germany), Regulatory affairs France: A. Ouslimani (Paris, France), **Treatment Manufacturing**: Sanofi-aventis (Antony, France), LC^2 (Lentilly, France), **Treatment management:** B. Lehmann, A. Tibi, Fabreguette (Paris, France), Cardinal (UK), Clindata (Germany)
Investigators within Countries
Principal Investigator (France/Germany/UK)

Centres (number of patients), Principal Investigators, Co-investigators (clinician/radiologist/psychologist)

France

Principal Investigator France: Y. Agid (Paris, France)

Aix en Provence (n = 20): F. Viallet, C. Couratier, S. Arguillère (clinicians), H. Payan-Cassin, G.M. Vassault (radiologists), P. Henon, S. Gimeno (psychologists); Angers (5): F. Dubas, C. Fressinaud (Clinicians), JY. Tanguy (radiologist), D. Legall (psychologist); Besançon (n = 7): L. Rumbach, E. Vidry (clinicians), J. Kraehenbuhl, JF. Bonneville (radiologists), G. Chopard (psychologist); Caen (n = 13): F. LeDoze, G. Defer, F. Viader, R-M. Marié (clinicians), H. Huet (radiologist), F. Daniel, C. Lalevée (psychologists); Clermont-Ferrand (n = 21): F. Durif, B. Debilly, Ph. Derost, C. Tilignac (clinicians), J. Gabrillargues (radiologist), D. Lauvergne Crégu, C. Rosière (psychologists); Grenoble (n = 7): G. Besson, C. Mallaret (clinicians), S. Grand (radiologist), H. Klinger, A. Funkiewiez (psychologists); Lille (n = 8): A. Destée, L. Defebvre (clinicians), C. Delmaire (radiologist), K. Dujardin (psychologist); Limoges (n = 12): P. Couratier (clinician), MP Boncoeur-Martel (radiologist), M. Chazot-Balcon (psychologists); Lyon (n = 11): E. Broussole, H. Mollion (clinicians), M Hermier (radiologist), M. Bouvard (psychologists); Marseille (n = 15): JP Azulay, T. Witjas (clinicians), (radiologist cf Aix en Provence), M. Delfini (psychologist); Montpellier (n = 16): W. Camu, F. Portet, J. Khoris, N. Pageot, G. Garrigues (clinicians), B. Viaud (radiologist), K. Martin, J. Bernard (psychologists); Nice (n = 8): M. Borg (clinician), S. Chanalet (radiologist), B. Bailet (psychologist); Paris patient clinical selection: M. Vidailhet, S. Sangla (Hôpital St Antoine), D. Ranoux (Hôpital St Anne), J.P. Brandel (Hôpital Leopold Belland), T. De Broucker (Hôpital St Denis), Y. Agid, B. Dubois, Meininger, Verny (Hopital Pitié-Salpêtrière), P. Cesaro (Hôpital Henri Mondor), G. Fenelon (Hôpital Tenon); Paris CIC Pitié-Salpêtrière-inclusion and follow-up (n = 111): Y. Agid, F. Bloch, A.M. Bonnet, L. Lacomblez, D. Maltête, A. Memin, Torni, ML. Welter, J. Worbe (Clinicians), T. Lalam, A. Tourbah, C. Marsault, Pr. D. Dormont (Radiologists), B. Pillon, V. Czernecki, A. Picard, B. Passaquet (psychologists); Pointe à Pitre (n = 5) D. Caparros-Lefebvre, A. Lannuzel (clinicians), (no radiologist), F. Verlut (psychologist); Poitiers (n = 12): R. Gil, M. Bailbé, S. Venisse, H. Moumy, V. Mesnage, J.L. Houeto, F. Petit (clinicians), P. Vandermarcq (radiologist), V. Bonnaud, C. Ornon (psychologists); Rennes (n = 13): M. Verin (clinician), Y. Rolland (radiologist), P. Trébon, G. Salicé (psychologists); Strasbourg (n = 16): C. Tranchant, G. Steinmetz (clinicians), JL Dietemann (radiologist), Crémel (psychologist); Toulouse (n = 8): O. Rascol, M. Galitzky, C. Thalamas (clinicians), P. Manelfe (radiologist), S. Lemoal, M.C. Deneuville, H. Delabaere (psychologists); Tours (n = 11): C. Prunier, A. Autret, P. Corsia (clinicians), P. Cottier, S. Gallas (radiologists), D. Beauchamp (psychologist).

Germany

Principal Investigator Germany: A. Ludolph (Ulm, Germany)

Aachen (n = 24): J. Noth, C. Kosinski, C. Geyer, M. Kronenbürger, C. Schlangen (clinicians), M. Doenges, S. Kémeny (radiologists), M. Kronenbürger (psychologist); Berlin (n = 37): K. Einhaeupl, PD G. Arnold, B. Hauptmann, A. Lipp (clinicians), A. Villringer, A. Lipp (radiologists), K. Fassdorf (psychologist); Bochum (n = 7): H. Przuntek, T. Müller, G. Gagel-Schweibold, M. Siepmann, S. Benz (clinicians), G. Schmid (radiologist), M. Finger, P. Klotz (psychologists); Dresden (n = 20): H. Reichmann, B. Herting (clinicians), R. von Kummer, D. Mucha (radiologists), E. Reissner (psychologist); Freiburg (n = 14): C. H. Lücking, I. Bötefür, S. Braune, C. Magerkurth, V. Mylius (clinicians), M. Schumacher, J. Spreer, S. Ziyeh (radiologists), C. Magerkurth, V. Mylius (psychologists); Halle (n = 7): S. Zierz, M. Kornhuber, T. Mueller, S. Neudecker, U. Seifert (clinicians), C. Behrmann, A. Schlueter (radiologists), A. Rockahr (psychologist); Hannover (n = 28): R. Dengler, A. Hauswedell, H. Kolbe, T. Peschel, C. Schrader, S. Siggelkow, J. Stewen, H.-H. Kapels, C. Winkler (clinicians), H. Heinze, G.

Kauffmann, M. Rotte (radiologists), C. Schrader (psychologist); Magdeburg (n = 7): C. W. Wallesch, C. Bartels, M. Fork (clinicians), S. Reissberg (radiologist), M. Fork (psychologist); München (n = 21): T. Brandt, F. Asmus, M. Bauer, T. Gasser, S. Maass, J. Velden, A. Viehöver, D. Wassilowsky, K. Bötzel (clinicians), T. Youssri, T. Wesemann, H. Brückmann, R. Brüning (radiologists), B. Baur, C. von Schlippenbach, G. Stenglein-Krapf (psychologists); Regensburg (n = 11): U. Bogdahn, J. Klucken, Z. Kohl, M. Lange, C. Thun, J. Winkler, B. Winner (clinicians), G. Schuierer (radiologist), M. Lange (psychologist); Rostock (n = 12): R. Benecke, D. Dressler, A. Wolters, G. Zegowitz (clinicians), G. Grau (radiologist), A. Fister (psychologist); Tübingen (n = 23): J. Dichgans, O. Eberhardt, K. Gröschel, T. K. Hauser, J. B. Schulz (clinicians), M. Skalej, T. K. Hauser (radiologists), T. K. Hauser (psychologist); Ulm (n = 31): A. C. Ludolph, D. Ecker, A. Jung, B. Kramer, G. B. Landwehrmeyer, A. Storch, S. D. Sussmuth (clinicians), E. Kraft, J. Kassubek (radiologists), I. Uttner (psychologist).

United Kingdom

Principal Investigator UK: PN Leigh (London, UK)

Belfast (n = 9): M. Gibson, R. Forbes (clinicians), C. Reynolds, C. S. McKinstry (radiologists), A. Dick (psychologist); Birmingham – City Hospital (n = 5): C. Clarke (clinician), S. Chavda (radiologist), S. Dhariwal, R. Hornabrook, S. Colhoun, V. Barnfield (psychologists); Birmingham – Queen Elizabeth Hospital (n = 9): H. Pall, D. Nicholls (clinicians), S. Chavda (radiologist), D. Nicholl (psychologist); Cambridge (n = 17): J. Hodges, T. Bak (clinicians), A. Carpenter (radiologist), T. Bak, V. Hearn, L. Donald (psychologists); Liverpool (n = 17): P. Moore (clinician), T. Dixon (radiologist), G. Baker, L. Owen, I. O'Brien (psychologists); London, King's College London (n = 55): P.N. Leigh, K. R. Chaudhuri, D. Heaney, C. Blain, S. Azam, V. Williams, J. Isaacs, C. Smallman, B. Stanton (clinicians), J. Jarosz, N. Deasey (radiologists), R. Brown, A. Dittner, C. Donnellan, D. Secker, A. Langman, C. Lomax (psychologists); C. Simeon (research nurse) London NHNN & Queen Square Hospital (n = 29): A. Lees, N. Quinn, A. Evans, T. Scaravilli, N. Russo, E. Trikouli, D. Paviour, Luke Massey (clinicians), C. Andrews, J. Stevens (radiologists), M. Jahanshahi, D. Winterburn (psychologists); Middlesborough (n = 7): P. Newman, Bathgate (clinicians), N. Bradey (radiologist), Z. Cowen (psychologist); Newcastle upon Tyne (n = 20): D. Burn, A. Zermansky, N. Warren (clinicians), P. English, A. Gholkar (radiologists), J. Welch, P. Welch (psychologists); Stafford (n = 9): B. Summers (clinician), D. Steventon (radiologist), B. Summers, L. Silver (psychologists); Aberdeen (n = 4): C. Counsell (clinician), A. Murray (radiologist), J. Gordon, C. Harris, K. Perkins (psychologists); Guernsey (n = 9) S. Bhaumick, S. Evans, G. Turner (clinicians), (no radiologist), S. Adam (psychologist); Swansea (14): R. Weiser, C. Lawthom, A. Lowman (clinicians), (no radiologist), G. Forwood, M. Moran, L. Bastin (psychologists).

Principal Investigator: P.N. Leigh (London, UK)

Co-ordination: European and UK: P.N. Leigh (London, UK), France: G. Bensimon (Paris, France), Germany: A.C. Ludolph (Ulm, Germany)

Steering Committee: Chair: P.N. Leigh (London, UK), Members: Y. Agid, G. Bensimon, M. Dib, L. Lacomblez, M. Vidailhet (Paris, France), D. Burn (Newcastle, UK); B. Landwehrmeyer, A.C. Ludolph (Ulm, Germany)

NNIPPS Investigators for France

Aix en Provence (n = 20): F. Viallet, C. Couratier, S. Arguillère (clinicians), H. Payan-Cassin, G.M. Vassault (radiologists), P. Henon, S. Gimeno (psychologists); Angers (5): F. Dubas, C. Fressinaud (Clinicians), JY. Tanguy (radiologist), D. Legall (psychologist); Besançon (n = 7): L. Rumbach, E. Vidry (clinicians), J. Kraehenbuhl, JF. Bonneville (radiologists), G. Chopard (psychologist); Caen (n = 13): F. LeDoze, G. Defer, F. Viader, R-M. Marié (clinicians), H. Huet (radiologist), F. Daniel, C. Lalevée (psychologists); Clermont-Ferrand (n = 21): F. Durif, B. Debilly, Ph. Derost, C. Tilignac (clinicians), J. Gabrillargues (radiologist), D. Lauvergne Crégu,

C. Rosière (psychologists); Grenoble (n = 7): G. Besson, C. Mallaret (clinicians), S. Grand (radiologist), H. Klinger, A. Funkiewiez (psychologists); Lille (n = 8): A. Destée, L. Defebvre (clinicians), C. Delmaire (radiologist), K. Dujardin (psychologist); Limoges (n = 12): P. Couratier (clinician), MP Boncoeur-Martel (radiologist), M. Chazot-Balcon (psychologists); Lyon (n = 11): E. Broussole, H. Mollion (clinicians), M Hermier (radiologist), M. Bouvard (psychologists); Marseille (n = 15): JP Azulay, T. Witjas (clinicians), (radiologist cf Aix en Provence), M. Delfini (psychologist); Montpellier (n = 16): W. Camu, F. Portet, J. Khoris, N. Pageot, G. Garrigues (clinicians), B. Viaud (radiologist), K. Martin, J. Bernard (psychologists); Nice (n = 8): M. Borg (clinician), S. Chanalet (radiologist), B. Bailet (psychologist); Paris patient clinical selection: M. Vidailhet, S. Sangla (Hôpital St Antoine), D. Ranoux (Hôpital St Anne), J.P. Brandel (Hôpital Leopold Belland), T. De Broucker (Hôpital St Denis), Y. Agid, B. Dubois, Meininger, Verny (Hopital Pitié-Salpêtrière), P. Cesaro (Hôpital Henri Mondor), G. Fenelon (Hôpital Tenon); Paris CIC Pitié-Salpêtrière-inclusion and follow-up (n = 111): Y. Agid, F. Bloch, A.M. Bonnet, L. Lacomblez, D. Maltête, A. Memin, Torni, ML. Welter, J. Worbe (Clinicians), T. Lalam, A. Tourbah, C. Marsault, Pr. D. Dormont (Radiologists), B. Pillon, V. Czernecki, A. Picard, B. Passaquet (psychologists); Pointe à Pitre (n = 5) D. Caparros-Lefebvre, A. Lannuzel (clinicians), (no radiologist), F. Verlut (psychologist); Poitiers (n = 12): R. Gil, M. Bailbé, S. Venisse, H. Moumy, V. Mesnage, J.L. Houeto, F. Petit (clinicians), P. Vandermarcq (radiologist), V. Bonnaud, C. Ornon (psychologists); Rennes (n = 13): M. Verin (clinician), Y. Rolland (radiologist), P. Trébon, G. Salicé (psychologists); Strasbourg (n = 16): C. Tranchant, G. Steinmetz (clinicians), JL Dietemann (radiologist), Crémel (psychologist); Toulouse (n = 8): O. Rascol, M. Galitzky, C. Thalamas (clinicians), P. Manelfe (radiologist), S. Lemoal, M.C. Deneuville, H. Delabaere (psychologists); Tours (n = 11): C. Prunier, A. Autret, P. Corsia (clinicians), P. Cottier, S. Gallas (radiologists), D. Beauchamp (psychologist).
